# Matrix-Free High-Resolution Atmospheric-Pressure SALDI Mass Spectrometry Imaging of Biological Samples Using Nanostructured DIUTHAME Membranes

**DOI:** 10.3390/metabo11090624

**Published:** 2021-09-15

**Authors:** Max A. Müller, Dhaka R. Bhandari, Bernhard Spengler

**Affiliations:** Institute of Inorganic and Analytical Chemistry, Justus Liebig University, 35392 Giessen, Germany; max.a.mueller@anorg.chemie.uni-giessen.de (M.A.M.); dhaka.r.bhandari@transmit.de (D.R.B.)

**Keywords:** mass spectrometry imaging, DIUTHAME, MALDI, SALDI, LDI, atmospheric pressure, high resolution

## Abstract

Applications of mass spectrometry imaging (MSI), especially matrix-assisted laser desorption/ionization (MALDI) in the life sciences are becoming increasingly focused on single cell analysis. With the latest instrumental developments, pixel sizes in the micrometer range can be obtained, leading to challenges in matrix application, where imperfections or inhomogeneities in the matrix layer can lead to misinterpretation of MS images. Thereby, the application of premanufactured, homogeneous ionization-assisting devices is a promising approach. Tissue sections were investigated using a matrix-free imaging technique (Desorption Ionization Using Through-Hole Alumina Membrane, DIUTHAME) based on premanufactured nanostructured membranes to be deposited on top of a tissue section, in comparison to the spray-coating of an organic matrix in a MALDI MSI approach. Atmospheric pressure MALDI MSI ion sources were coupled to orbital trapping mass spectrometers. MS signals obtained by the different ionization techniques were annotated using accurate-mass-based database research. Compared to MALDI MSI, DIUTHAME MS images captivated with higher signal homogeneities, higher contrast and reduced background signals, while signal intensities were reduced by about one order of magnitude, independent of analyte class. DIUTHAME membranes, being applicable only on tissue sections thicker than 50 µm, were successfully used for mammal, insect and plant tissue with a high lateral resolution down to 5 µm.

## 1. Introduction

Mass spectrometry imaging (MSI) has proven to be a valuable and versatile tool for spatially resolved chemical analysis of surfaces [1,2,3]. Particularly, matrix-assisted laser desorption/ionization (MALDI) MSI [4,5] under atmospheric pressure (AP) is known for its ease of sample handling, morphological authenticity and the possible combination with highly accurate, highly mass resolving Fourier-transformation-based mass spectrometers [3,6,7]. A key characteristic is the achievable lateral resolution for a detailed examination of biological structures.

With recent advancements in AP-MALDI MSI, it became possible to achieve subcellular lateral resolution down to 1.4 µm per pixel and simultaneously accomplish a mass resolution of >100,000 in less than one second using orbital trapping mass spectrometers [8]. The achievable lateral resolution is predominantly defined by the focal diameter of the laser beam on the sample surface and thereby the sample area from which analytes are desorbed and ionized. For small laser spot sizes, a high ion yield is essential, predominantly influenced by the chosen matrix material [8]. Application of the matrix, however, becomes more and more challenging with increasing lateral resolution, since inhomogeneities in the matrix layer and matrix crystals larger than the expected lateral resolution almost always have a negative impact [9]. Optimizing protocols for matrix application to achieve homogeneous surface coverage and small matrix crystals while retaining high ion yields is crucial but time-consuming, as it relies on many parameters [10]. Introducing new matrices for high lateral resolution is therefore challenging.

A variety of organic acids and bases can be used as matrices in positive- or negative-ion mode, respectively [11,12]. Most importantly, a matrix should have optimal spectral absorption characteristics at the wavelength of the employed laser, be inert to oxidation and non-reactive with the sample, and should generate minimal background signal, a major challenge especially for low molecular mass analytes [2,10,13].

In contrast, desorption electrospray ionization (DESI) MSI is a widely used matrix-free imaging technique that uses solvents instead of laser radiation as a sample probe and thereby is reducing sample preparation time and effort compared to MALDI MSI [14,15]. Despite its versatility and ease of operation, DESI MSI cannot yet reach the high lateral resolution commonly achieved by MALDI MSI [16].

To obtain high lateral resolution without the need for a matrix to absorb the specific laser wavelength, it has been demonstrated that nanostructured surfaces can also assist in desorption and ionization (surface assisted laser desorption/ionization, SALDI) [17,18]. Despite the mechanism being not well defined, a variety of materials such as carbon [17,19,20], silicon [21,22], metals [23,24,25,26], or organic surface coatings [27] with different nanostructures have also been tested for imaging applications. Among such materials, DIUTHAME (Desorption Ionization Using Through-Hole Alumina Membrane) is showing promising features for an easy and reproducible sample preparation [28,29,30,31]. DIUTHAME consists of a 5 µm thin alumina membrane, nanostructured with 200 nm through holes. As it is manufactured in an automated process before any contact with samples, inhomogeneities are not as common as for MALDI sample preparations, where a matrix is applied directly onto the tissue. This makes DIUTHAME a promising candidate for high lateral resolution MSI [28]. It has been shown that analytes such as lipids, peptides, or small proteins can be desorbed and ionized by laser irradiation of DIUTHAME samples of standard solutions in time-of-flight (TOF) mass spectrometers under vacuum conditions with high reproducibility [28]. Under these conditions, Kuwata et al. were able to perform MSI experiments with a lateral resolution of 50 µm and a mass resolution of up to 50,000 from mouse brain tissue [30].

Here, we investigated the performance and characteristics of DIUTHAME for a higher lateral resolution of 5 µm at high mass resolution (up to 240,000) using atmospheric-pressure MSI of biomolecules from native tissues from different organisms. We evaluated the performance regarding the detectable analyte classes, sensitivity, achievable lateral resolution, and image quality in comparison to MALDI and LDI experiments.

## 2. Results

### 2.1. Desorption and Ionization Using DIUTHAME

To characterize the ionization behavior of DIUTHAME, mouse brain tissue sections were analyzed and compared to the results obtained from MALDI and LDI MSI experiments. For comparison, the ablation spot size was kept constant at 5 µm diameter, corresponding to an ablation spot size of ≈20 µm^2^ (Figure S1); therefore, laser energy had to be optimized for each ionization method individually. While LDI without any tissue pretreatment had to be performed with a very high laser fluence (≈500 kJ/m^2^) to yield significant signal intensities and visible ablation spots on the sample, DIUTHAME and MALDI could be performed with much lower laser pulse energies of ≈1300 J/m^2^ and ≈2500 J/m^2^, respectively.

**Figure 1:**
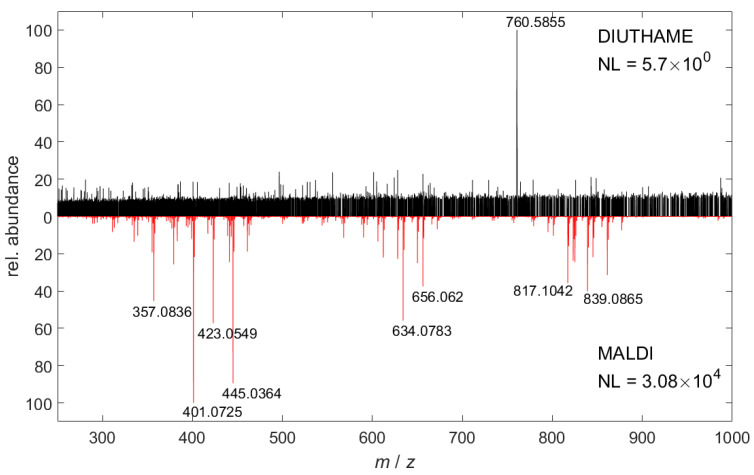
Comparison of 100 summed-up mass spectra in positive-ion mode acquired by DIUTHAME MS (black) from blank DIUTHAME membrane and MALDI MS (red) from pure CHCA matrix on glass, respectively.

Blank spectra of pure matrix or DIUTHAME membranes without applying analytes differed significantly (Figure 1). Since the MALDI matrix itself is desorbed and ionized, it produces characteristic signals in the mass spectrum. From blank (Figure 1) or incompletely attached DIUTHAME foils (Figures S2 and S3), no significant signals were observed, an observation typical for SALDI MS [18,32]. Compound identification and quantification take advantage of background-free spectra in (partly) overlapping areas of spectra, even under high mass resolution conditions.

The results show that the DIUTHAME foils during desorption and ionization work similarly efficient as the matrix in the MALDI process, leading to comparable laser energy settings, very different from LDI conditions from solid sample supports. Since the DIUTHAME material itself is not ionized, it is clear, on the other hand, that the mechanisms of energy uptake, analyte extraction, desorption and ionization are very different in nano-structured sample supports compared to matrix microcrystals.

### 2.2. Signal Quality and Quantity for DIUTHAME

**Figure 2:**
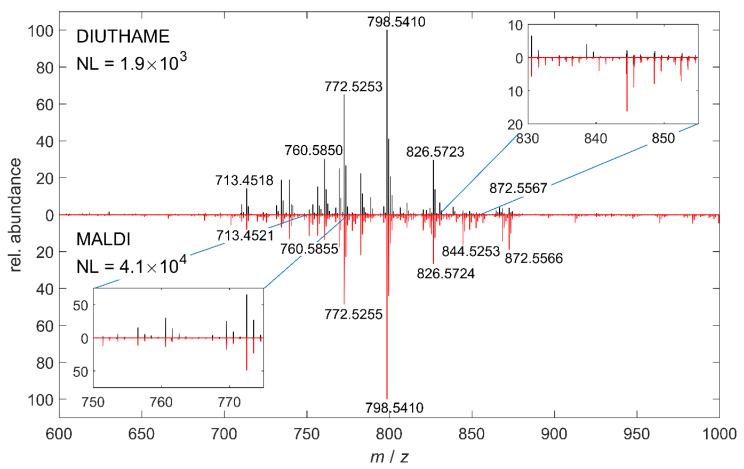
Comparison of 100 summed mass spectra acquired by DIUTHAME (black) and MALDI (red) MSI, respectively, from adjacent mouse brain tissue sections in the cerebellum region in positive-ion mode.

In our MSI setup, desorption and ionization of analytes from biological tissue was possible with DIUTHAME in positive-ion mode. On mouse-brain tissue, signal intensities (normalized level NL) of the DIUTHAME measurements were lower by one order of magnitude compared to MALDI with the CHCA matrix (Figure 2).

**Figure 3:**
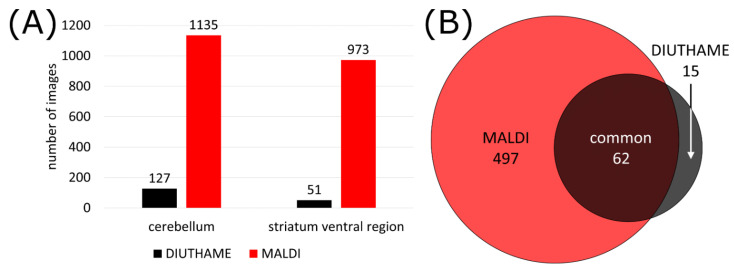
(**A**) Number of images available from mouse brain tissue with DIUTHAME and MALDI MSI, respectively. Only phospholipid signals in a mass range between *m*/*z* 600–1000 with a pixel coverage of >5% were considered, matrix signals were excluded. (**B**) Venn diagram of annotated phospholipids from signals detected with a MALDI or DIUTHAME MSI measurement.

As a result, fewer analytes reached the limit of detection, and in MSI experiments, a smaller number of images were generated from untargeted measurements. In total, 1135 versus 127 signals in the phospholipid mass range (*m*/*z* 600–1000, decimal place 0.4–0.7) were present in at least 5% of all pixels for MALDI and DIUTHAME, respectively (Figure 3A), from a mouse brain cerebellum. Similar behavior was observed for the striatum ventral region of the mouse brain. A database search resulted in phospholipid annotations for 559 (49%) and 77 (61%) of these signals for MALDI and DIUTHAME, respectively (Tables S1 and S2). The comparison revealed, that the annotations of DIUTHAME signals were mostly a subset of those found with MALDI (Figure 3B). Of all distinct phospholipid annotations, 497 (86%) were found exclusively in MALDI measurements, 15 (3%) exclusively in DIUTHAME measurements, and 62 (11%) were shared between the two.

Protonated signals were more prominent in the mass spectra when ionizing via DIUTHAME (30% of number of signals) compared to MALDI (25% of number of signals), whereas MALDI mass spectra had a higher percentage of [M + K]^+^ adducts (41%) compared to DIUTHAME (35%). The fraction of [M + Na]^+^ species was comparable between DIUTHAME (35%) and MALDI (34%) (Figure S4).

Lipid classes detected in positive-ion mode were comparable for DIUTHAME and MALDI, as both techniques preferably ionize phosphatidylcholine and phosphatidylethanolamine species (Figure S4). The percentage of phosphatidylserine, phosphatidylglycerol, or phosphatic acid lipid species were comparable. None of the lipid classes were observed exclusively by one of the ionization techniques. Nevertheless, MALDI showed more of those lipid signals, which do not fit into standard categories, making the signals obtained by MALDI more diverse compared to DIUTHAME. This is most probably due to the overall lower signal intensities in DIUTHAME measurements, where hard-to-ionize and lower-abundant analyte species remain below the limit of detection.

In negative-ion mode, however, DIUTHAME did not generate any ion signal of analytes from mouse brain or mouse kidney tissue. The resulting mass spectra were comparable to blank DIUTHAME instead (Figure S5). In the literature, mostly positive-ion spectra were shown for DIUTHAME MSI experiments [28,30]. Negative-ion-mode experiments were only shown for time-of-flight (TOF) instruments with significantly reduced signal intensities compared to positive-ion mode [31]. The reason for this behavior might be that DIUTHAME, in contrast to MALDI matrices, does not introduce charge carriers into the rather acidic biological system but is only providing an active surface. In combination with the overall lower signal intensities from DIUTHAME compared to MALDI in our MSI setup, the number of ions generated from DIUTHAME in negative-ion mode appears to be below the detection threshold. In the lower mass range (*m*/*z* 250–500), DIUTHAME did not produce reliable signals from mouse kidney tissue (Figure S6), making it not suitable for the analysis of small metabolites. Additionally, no other biological species except lipids (such as peptides or protein fragments) could be detected (based on mass-defect calculations) from mouse brain or mouse kidney tissue.

### 2.3. MSI of Biological Tissues Using DIUTHAME, MALDI and LDI

As DIUTHAME is capable of retaining the spatial information of analytes within the tissue section, it can be used in MS imaging experiments. To characterize its performance, mouse brain tissue sections were analyzed using DIUTHAME, MALDI and LDI MS imaging techniques in comparison. MALDI was selected as a widely used method for MS imaging. The LDI experiments were performed to check if the DIUTHAME nanostructures improve the ionization yield over direct laser desorption/ionization from solid surfaces.

Comparable mouse brain regions in the cerebellum and the striatum ventral region from consecutive sections were investigated by DIUTHAME, MALDI and LDI for an area of 300 × 250 pixels with a pixel size and laser spot diameter of 5 µm. While the employed experimental setup is capable of focusing the laser to a smaller spot size [8], 5 µm was the smallest laser spot diameter yielding sufficient ion signal intensities for DIUTHAME imaging experiments due to its lower ionization efficiency as mentioned earlier. Signals in the phospholipid mass range of *m*/*z* 600–1000 were recorded in positive-ion mode.

The experiments clearly show, that LDI from biological tissue without the assistance of matrix or an ionizing nanostructured membrane results in very low signal intensities, poor image quality and many blank pixels below the detection threshold, clearly indicating that DIUTHAME similar to MALDI is playing an important role in the desorption and ionization process. Further, a high noise level in mass spectra and images, resulting from strong background ionization at high laser power, was observed, making it tough to find signals representing anatomical structures in the tissue investigated (Figure S7).

When comparing DIUTHAME and MALDI, it became apparent that both techniques generate similar image quality. Displaying the same *m*/*z* signals, both measurement techniques clearly show white matter, grey matter and a granular layer in the cerebellum region. Purkinje cells were spotted by the absence of the surrounding signals between the granular layer and white matter. In MALDI MSI, distinct marker signals were detected representing the Purkinje cells (Figure S8), while these were not found in DIUTHAME MSI. In the striatum ventral region, both ionization techniques precisely outlined the small spots of interlaced lateral globus pallidus. The adjacent microscopic images corresponded well, even though for the DIUTHAME application the optical images were generated with the membrane attached, leading to a rather poor quality at high magnification.

**Figure 4:**
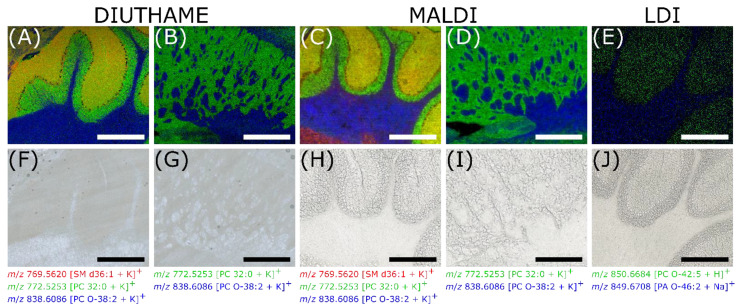
Comparison of DIUTHAME, MALDI and LDI MSI from adjacent mouse brain tissue sections, acquired with a pixel size of 5 µm, an image size of 300 × 250 pixels, a laser focal diameter of 5 µm, in a mass range of m/z 600–1000. (**A**), (**F**), DIUTHAME MS image of mouse brain cerebellum with corresponding microscopic image. (**B**,**G**), DIUTHAME MS image of mouse brain striatum ventral region with corresponding microscopic image. (**C**,**H**), MALDI MS image of mouse brain cerebellum with corresponding microscopic image. (**D**,**I**), MALDI MS image of mouse brain striatum ventral region with corresponding microscopic image. (**E**,**J**), LDI MS image of mouse brain cerebellum with corresponding microscopic image. Thickness of tissue sections (**A**,**B**), 50 µm, (**C**–**E**), 20 µm. Scale bars: 500 µm.

DIUTHAME, compared to MALDI, showed a slight increase in sharpness and contrast of the MS images, as well as an increased signal homogeneity in uniform tissue regions (Figure 4). This is due to the fact that sample preparation is becoming a crucial step in MALDI at small pixel sizes. Slight inhomogeneities during the application of the matrix become more apparent, and washout effects result in a minor image blur due to solvent use. Solvent-free matrix application methods such as dry sublimation usually result in a lower ion yield due to lacking co-crystallization of matrix and analyte [33]. Performing MSI with DIUTHAME also does not involve solvent use, but the moisture of the tissue itself is usually sufficient to induce analyte uptake into the membrane while preventing washout effects. More *m*/*z* images generated from the DIUTHAME MSI are shown in Figure S9.

### 2.4. DIUTHAME MSI of Tissue Sections from Various Organisms

In addition to mouse brain tissue sections, DIUTHAME MSI was tested on a variety of sample types from different biological species and tissues such as a mouse kidney section (276 × 161 pixels, 30 µm pixel size, full-pixel mode [34]), a germinating rapeseed section (297 × 245 pixels, 20 µm pixel size) and a *Spodoptera littoralis* (caterpillar) section (300 × 250 pixels, 20 µm pixel size).

**Figure 5:**
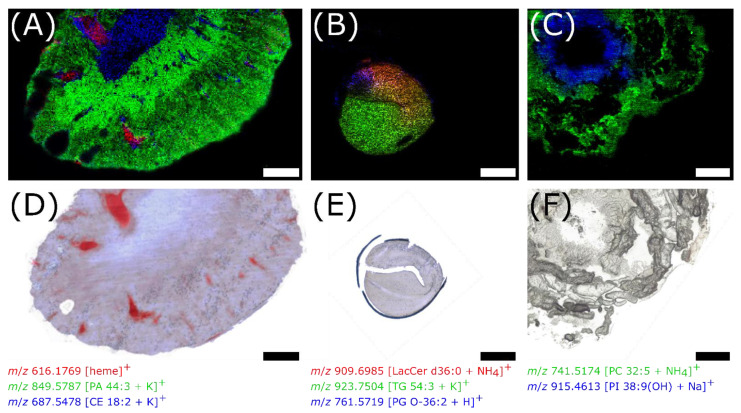
MS images of tissue sections from various organisms using DIUTHAME as the ionization assisting membrane (**A**–**C**) and corresponding microscopic images of the same (**E**) or an adjacent section (**D**,**F**). (**A**), 50 µm thick mouse kidney section, pixel size 30 µm, 276 × 161 pixels, *m*/*z* 400–1600. (**B**), 80 µm thick germinating rapeseed section, pixel size 20 µm, 297 × 245 pixels, *m*/*z* 500–1500. (**C**), 150 µm thick *Spodoptera littoralis* caterpillar section, pixel size 20 µm, 300 × 250 pixels, *m*/*z* 250–1000. Scale bars: 1 mm.

In the mouse kidney section, distinct signals of phospholipids were detected in the medulla and cortex as well as a signal deriving from the heme group in blood vessels (Figure 5A). In germinating rapeseed sections, various triglycerides [35] were detected in the endosperm as previously described for MALDI MSI [36], as well as growth-state-dependent phospholipids in the root tip (Figure 5B). However, comparing experiments from the same study, only half of the signals detected with MALDI, including phospholipids, diglycerides, triglycerides, or spermidine conjugates, were annotated in the seed sections of the rapeseed plant using DIUTHAME [36]. This is due to the overall lower intensities and sensitivity of DIUTHAME compared to MALDI, resulting in more analytes remaining below the limit of detection. For the *Spodoptera littoralis* larva section, phospholipid signals nicely outline the caterpillar’s body and the gut wall (Figure 5C). Additional MS images are shown in Figures S10–S12.

The experiments show the capability of DIUTHAME to produce highly resolved MS images with high contrast, expressing detailed anatomical features, independent of the biological origin of the sample. The technique has been found to be feasible on soft (mouse brain, mouse kidney), hard (rapeseed), or fragile (larva) tissue sections from mammals, plants, or insects. As shown for the mouse brain earlier, not only can tissue regions be clearly distinguished, but also fine structures and gradients can be determined as shown for the rapeseed section. Therefore, the signal intensity of *m*/*z* 909.6985 was plotted against its spatial position along the growth direction of the rapeseed (Figure S13), indicating a gradual enrichment of the compound towards the root tip.

## 3. Discussion

While SALDI MSI applications are an emerging field of research, little is known about the underlying mechanisms of desorption and ionization [37]. For SALDI, employing a variety of mostly inorganic materials with different shapes, sizes, or nanostructures, elucidation of these mechanisms is an ongoing matter of debate. In general, it is presumed that thermal and non-thermal processes are involved in the process of desorption and ionization [18,32,38].

One of the main roles of the nanomaterial is to absorb the energy of the ionization laser, resulting in a rapid and spatially confined increase in surface temperature, assisting analytes to desorb from the surface. In the case of DIUTHAME, this is amplified by the fact that analytes are confined into nanocapillaries. Nevertheless, recent studies using thermometer molecules revealed that not only thermal desorption is the main aspect in SALDI, but that also phase transitions play a major role. It was observed that signal intensities of analytes sharply increased when the energy input by the laser reached a phase transition threshold [39]. Mechanistic investigations were not the main goal of our study. It was observed, however, that desorption and ionization with DIUTHAME required laser energies high enough to produce visible ablation marks on the membrane (Figures S1 and S14), which supports the idea of nanomaterial destruction and phase transitions being involved in the process. Laser energy thresholds for efficient desorption are a possible obstacle on further improving the lateral resolution of DIUTHAME MSI measurements, since high lateral resolution is usually linked to lower laser energy settings [8].

The process of ionization, being assisted by nanomaterials, is even less understood [37]. Charge carriers have to be either already present in the sample or transferred to the analyte from the nanomaterial, whereas the mechanism of the latter is highly debated. For example, one mechanism involves high-energy electrons, so-called hot electrons, being ejected by the nanomaterial upon laser irradiation and subsequent rapid heating [40]. Analytes can either use these ejected electrons or, even more efficiently, the remaining electron holes [41] in the nanomaterial for ionization in positive- or negative-ion mode, respectively. This charge accumulation in the nanomaterial would also lead to Coulomb explosion of the material [42], producing charged nanomaterial clusters, that should be detectable in the mass spectrometer [37]. Nevertheless, in our experiments with DIUTHAME, no such clusters were detected (Figure 1), presumably speaking against charge carriers to be ejected from the nanomaterial or being transferred to the analyte in significant quantities. Additionally, annotation of signals by accurate mass measurements revealed a high number of normal quasimolecular ions, such as proton, sodium or potassium adducts (Figure S4), that cannot originate from the DIUTHAME material and must have been pre-existing in the sample or produced by photochemical interactions of the laser with the water of the samples [37].

Due to the complexity and variability of nanomaterials, the underlying desorption and ionization mechanism is hard to elucidate. Further, this hinders targeted improvements regarding desorption and ionization efficiency [43], which in the case of DIUTHAME could be helpful to overcome the lower sensitivity compared to MALDI MSI and to potentially enable even higher lateral resolution measurements.

## 4. Materials and Methods

### 4.1. MSI Instrumentation

MSI measurements were carried out on an AP-SMALDI5 AF ion source (TransMIT GmbH, Giessen, Germany) coupled to a ‘Q Exactive HF’ orbital trapping mass spectrometer (Thermo Fisher Scientific GmbH, Bremen, Germany) and alternatively on a home-built ultra-high-resolution AP-MALDI MSI source, coupled to a ‘Q Exactive’ orbital trapping mass spectrometer (Thermo Fisher Scientific GmbH, Bremen, Germany). Laser fluence was adjusted by controllable dichroic filters. The highest available mass resolution of 240,000 or 140,000 was used for all experiments on the Q Exactive HF or Q Exactive, respectively. A fixed injection time of 500 ms was set on both devices and a high voltage of 4 kV was applied to the samples. Laser energy was adjusted individually for each experiment.

### 4.2. Sample Preparation

**Figure 6:**
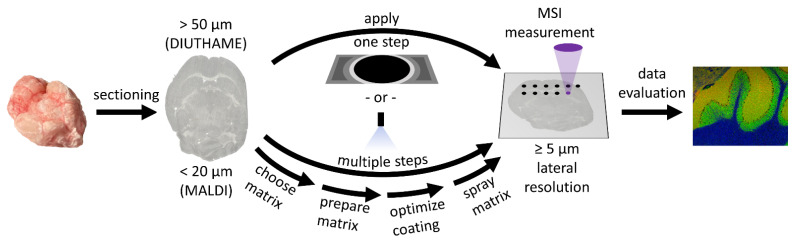
Illustrative overview of the workflow for a DIUTHAME and a MALDI MSI measurement, respectively. The main difference in the workflow is the preparation step between sectioning and MSI measurement.

Thin tissue sections from fresh-frozen tissue were prepared using a microcryotome (Microm HM 525, Thermo Fisher Scientific GmbH, Bremen, Germany) at −20 °C. Tissue sections with a thickness of 20 µm were chosen for MALDI and LDI measurements, whereas for experiments with DIUTHAME, tissue sections with a thickness of 50–150 µm were used (Figure 6).

DIUTHAME-ionizing membranes (Hamamatsu Photonics, Hamamatsu, Japan) have a circular effective area of up to 18 mm in diameter, fixed in a metal frame with a self-adhesive backside (Figure S14). After exposing the adhesive area, the effective area of the membrane has to be placed on top of a frozen tissue section without application of pressure (Figure 6). Due to the low mechanical strength of the 5 µm thin membrane, even weak forces applied can break the membrane and therefore render it useless for further experiments. During thawing of the sample, the membrane attaches itself to the tissue in a process similar to thaw-mounting. As a result of the geometry of the frame-membrane arrangement of the first-generation DIUTHAME units, tissue sections had to be at least 50 µm thick (Figure S2) to ensure a firm and complete attachment of the membrane without causing air bubbles, which would lead to a loss of signal and blind spots in MSI experiments (Figure S3). For dry tissue sections such as germinated seeds from a rapeseed plant, this process was facilitated by applying 2 µL of ethanol on top of the already attached membrane.

For MALDI measurements, matrix was applied using a SMALDIPrep (TransMIT GmbH, Giessen, Germany) pneumatic spraying system. A total of 14 mg of α-Cyano-4-hydroxycinnamic acid (CHCA, Sigma Aldrich, Munich, Germany) were dissolved in 1998 µL of a mixture of 1:1 acetone-water and acidified with 2 µL of pure trifluoroacetic acid (Merck KGaA, Darmstadt, Germany) to produce 2 mL of a solution with a matrix concentration of 7 mg/mL. A volume of 80 µL of the solution was applied to the sample by pneumatic spraying at a flow rate of 10 µL/min (Figure 6).

### 4.3. Histology

Microscopic images were acquired with a digital microscope (VHX-5000, Keyence GmbH, Neu Isenburg, Germany) before the MSI experiment under epi-illumination for MALDI and LDI samples. Precedent to DIUTHAME experiments, the microscopic images were acquired with transmitted light with the membrane attached, since it has to be applied prior to thawing (Figure S15).

After MALDI MSI experiments, the matrix was removed with ethanol and the tissue was stained with hematoxylin and eosin (Figure S16) following an established protocol (Table S3). Histological staining is not possible after a DIUTHAME experiment since the membrane cannot be removed from the tissue.

### 4.4. Data Analysis

Mass spectra from mouse brain tissue were recalibrated to *m*/*z* 798.5410, which is known to be [PC 34:1 + K]^+^. MALDI images were created using Mirion [44] software with a bin width ∆(*m*/*z*) of ± 0.005, and signal intensities were normalized to total ion charge [34] in each single pixel. Signals were evaluated if they were detected in at least 5% of the pixels of an image. The signal assignment was carried out via a database search in LIPID MAPS [45] using compiled data from up to 75,000 mass spectra per experiment. Potential lipid groups and ion species were assigned based on the smallest deviations from calculated *m*/*z* values and allowing for a maximal mass error of 3 ppm without cross-validating for isotopologues or different ion adducts being present and showing the same lateral distribution. Thereby, annotations remain putative. All data from imaging experiments were uploaded to metaspace [46], a platform for metabolite annotation of MSI data. The results from the platform are publicly available.

## 5. Conclusions

We demonstrated the applicability of DIUTHAME membranes for MSI at a high lateral resolution of 5 µm pixel size under atmospheric-pressure conditions. Due to DIUTHAME assisting desorption and ionization via a SALDI-like mechanism, background signals are reduced compared to MALDI or LDI experiments. MS images produced with DIUTHAME benefit from a higher signal homogeneity and a higher contrast than those produced by MALDI experiments under otherwise same conditions. Tissue sections for DIUTHAME experiments have to be significantly thicker (≥50 µm) than for MALDI (≤20 µm) for geometrical reasons. Tissue sections from different organisms including mammals, insects and plants were successfully investigated, and various analyte classes such as metabolites (*m*/*z* > 500), triglycerides, phospholipids, or enzymatic co-factors were detected. However, the ionization efficiency of DIUTHAME is significantly lower than that of MALDI, resulting in signal intensities being reduced by roughly one order of magnitude for DIUTHAME, thus hindering the detection of lowly abundant or hard-to-ionize analytes (e.g., metabolites (*m*/*z* < 500), drugs, peptides) which are nicely detectable by MALDI MSI. Thereby, DIUTHAME cannot always be used to improve MSI at higher lateral resolution yet, but might be improved in the future in terms of thinner tissue sections and higher ionization efficiencies.

## Data Availability

Data are publicly available on metaspace, an annotation platform for mass spectrometry imaging under the following link: https://metaspace2020.eu/project/diuthame_mueller (accessed on 1 September 2021).

## Supplementary Materials

The following are available online at https://www.mdpi.com/article/10.3390/metabo11090624/s1, Figure S1: Microscopic image of ablation spots in MALDI, DIUTHAME and LDI mode, Figure S2: Scheme of incomplete or complete DIUTHAME attachment, Figure S3: MS image from incompletely attached DIUTHAME on mouse brain tissue, Figure S4: Pie chart of ion adducts and lipid class annotations for MS measurements with MALDI or DIUTHAME on mouse brain tissue, Figure S5: Mass spectra from blank DIUTHAME and DIUTHAME attached to mouse kidney tissue in negative-ion mode, Figure S6: Mass spectrum of DIUTHAME measurement on mouse kidney tissue with a mass range from *m*/*z* 250–1000, Figure S7: Mass spectrum of LDI measurement on mouse brain tissue, Figure S8: MALDI MS image from mouse brain cerebellum showing Purkinje cells, Figure S9: MS images from mouse brain tissue with DIUTHAME membrane attached, Figure S10: MS images from mouse kidney tissue with DIUTHAME membrane attached, Figure S11: MS images from germinated rapeseed with DIUTHAME membrane attached, Figure S12: MS images from *Spodoptera littoralis* tissue with DIUTHAME membrane attached, Figure S13: Evaluation of intensity gradient from rapeseed DIUTHAME MSI measurement, Figure S14: Optical image of a DIUTHAME membrane, Figure S15: Microscopic image of mouse brain tissue with a DIUTHAME foil attached, Figure S16: Microscopic image of mouse brain tissue stained with hematoxylin and eosin, Table S1: List of lipid annotations to mass signals for DIUTHAME MSI on mouse brain tissue, Table S2: List of lipid annotations to mass signals for MALDI MSI on mouse brain tissue, Table S3: Protocol for hematoxylin and eosin staining.
