# Supplementary material for: Matrix-Free High-Resolution Atmospheric-Pressure SALDI Mass Spectrometry Imaging of Biological Samples Using Nanostructured DIUTHAME Membranes
Source: Metabolites. 2021 Sep 15;11(9):624. doi: 10.3390/metabo11090624 (PMC8468348; doi:10.3390/metabo11090624)
Supplement: Supplementary file 1 [file metabolites-11-00624-s001.zip › metabolites-1322067-supplementary.pdf]

Matrix-free high-resolution atmospheric-pressure SALDI mass spectrometry imaging of biological samples using DIUTHAME membranes

Max A. Müller, Dhaka R. Bhandari, Bernhard Spengler

Institute of Inorganic and Analytical Chemistry, Justus Liebig University, Giessen, Germany

Supporting Information

		page
1.	Microscopic image of ablation spots in MALDI, DIUTHAME and LDI mode	2
2.	Scheme of incomplete or complete DIUTHAME attachment	2
3.	MS image from incompletely attached DIUTHAME on mouse brain tissue	2
4.	Pie charts of ion adducts and lipid class annotations for MS measurements with MALDI or DIUTHAME on mouse brain tissue	3
5.	Mass spectra from blank DIUTHAME and DIUTHAME, attached to mouse kidney tissue in negative-ion mode	4
6.	Mass spectrum of DIUTHAME measurement on mouse kidney tissue with a mass range from *m/z* 250-1000	4
7.	Mass spectrum of LDI measurement on mouse brain tissue	5
8.	MALDI MS image from mouse brain cerebellum showing Purkinje cells	5
9.	MS images from mouse brain tissue with DIUTHAME membrane attached	6
10.	MS images from mouse kidney tissue with DIUTHAME membrane attached	7
11.	MS images from germinated rapeseed with DIUTHAME membrane attached	8
12.	MS images from *Spodoptera littoralis* tissue with DIUTHAME membrane attached	9
13.	Evaluation of intensity gradient from rapeseed DIUTHAME MSI measurement	10
14.	Optical image of DIUTHAME membrane	10
15.	Microscopic image of mouse brain tissue with DIUTHAME membrane attached	11
16.	Microscopic image of mouse brain tissue stained with hematoxylin and eosin	12
17.	List of lipid annotations to mass signals for DIUTHAME MSI on mouse brain tissue	13
18.	List of lipid annotations to mass signals for MALDI MSI on mouse brain tissue	15
19.	Protocol for hematoxylin and eosin staining	27
20.	References for the Supporting Information	27

*Address correspondence to:
Institute of Inorganic and Analytical Chemistry
Justus Liebig University Giessen
Heinrich Buff Ring 17
35392 Giessen, Germany
Phone: +49 641 99 34801
e-mail: bernhard.spengler@anorg.chemie.uni-giessen.de

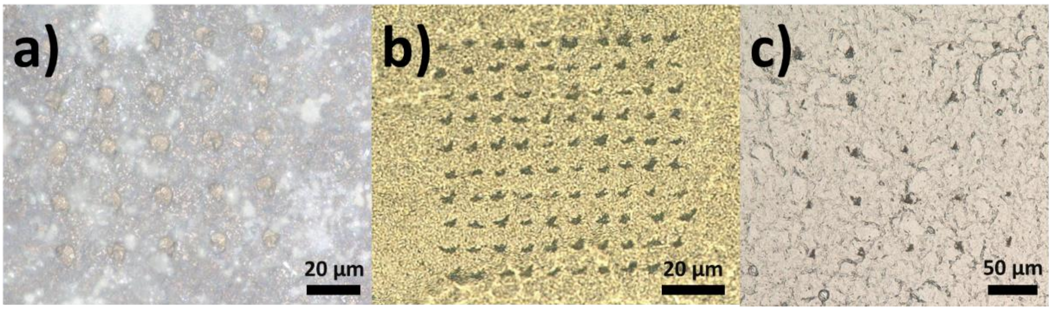

Supporting Figure 1: Microscopic images of ablation spots produced by MALD MSI with 20 µm step size (a), DIUTHAME with 10 µm step size (b) and LDI with 50 µm step size (c), respectively. Laser intensity was the same as for the imaging experiments shown in Figure 2.

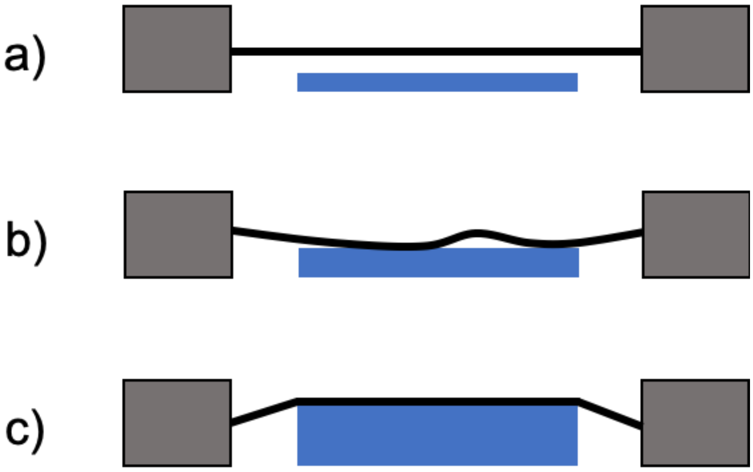

Supporting Figure 2: Scheme of DIUTHAME effective area (black) attachment to a sample (blue). a) No connection between DIUTHAME and the sample when the sample is too thin. Thereby, no ion signal is generated. b) Incomplete attachment of DIUTHAME to a medium thick sample, leaving room for air bubbles, causing intermediate signal loss. c) Complete and firm attachment of DIUTHAME to a thicker sample, ensuring a homogeneous and distortion-free desorption and ionization of analytes.

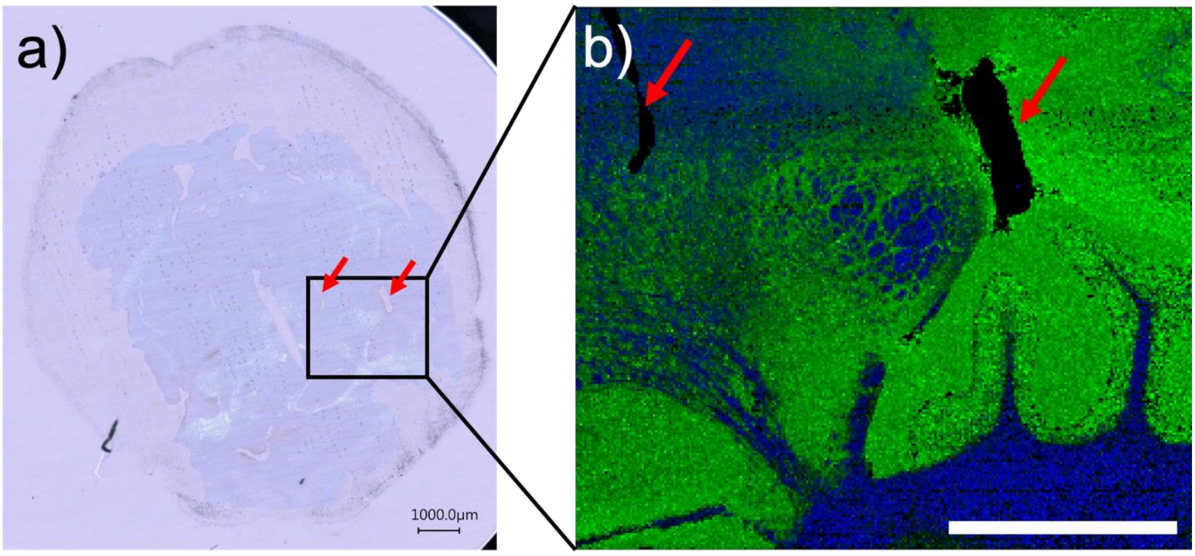

Supporting Figure 3: a) Microscopic image of mouse brain tissue with incomplete DIUTHAME membrane attachment. Air bubbles are highlighted with red arrows. b) DIUTHAME MSI image of a region in mouse brain cerebellum in positive-ion mode. Pixel size 10 µm, 270x270 pixels, m/z 600 – 1000. Color coding: green: m/z 772.5279 [PC 32:0 + K]$^+$, blue: m/z 848.6405 [PS 40:0 + H]$^+$. Red arrows indicate the position of the air bubbles visible from (a), producing blind spots in the MSI results. Scale bars: 1 mm.

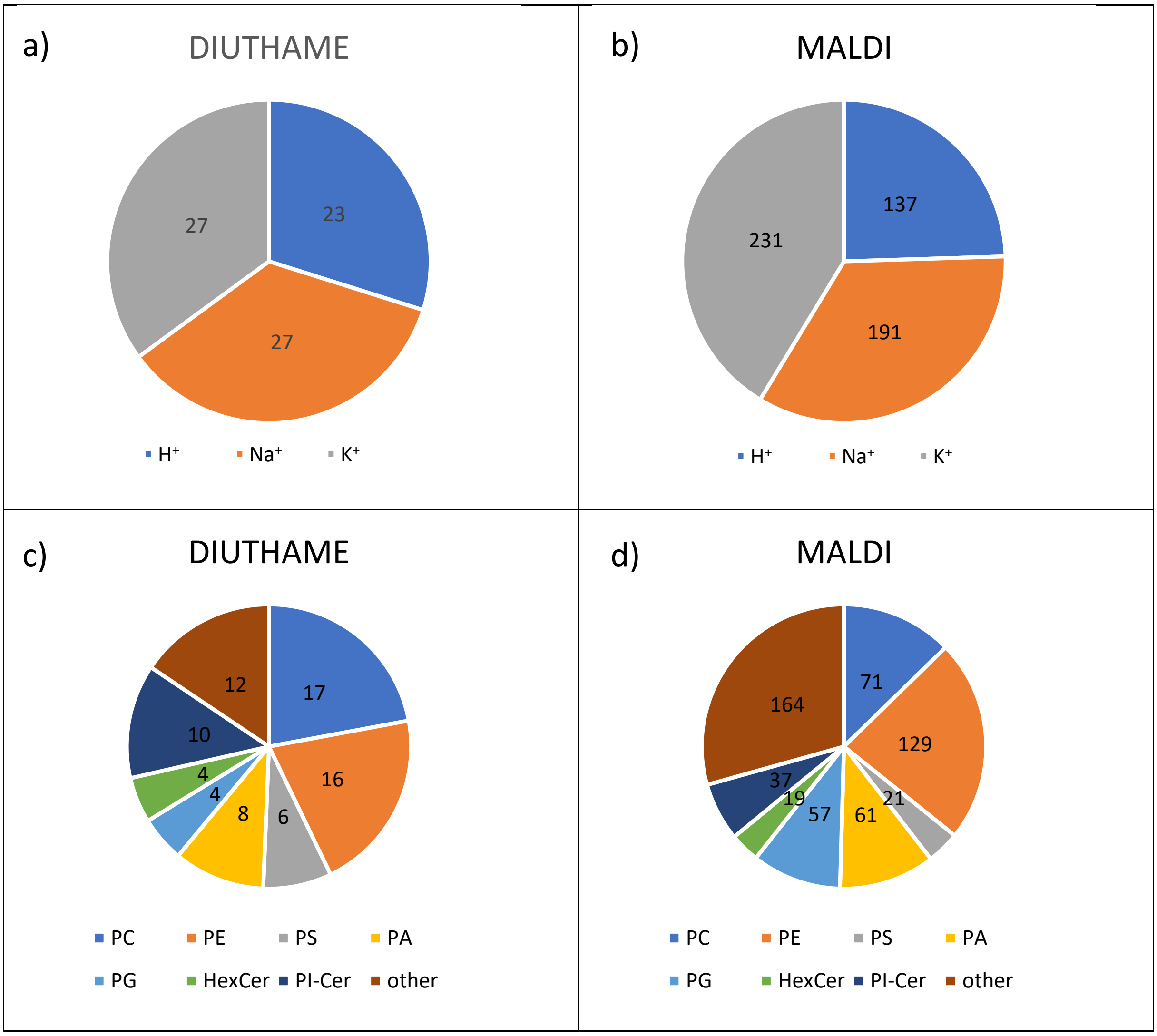

Supporting Figure 4: Pie chart of the ion adducts and lipid classes found through annotation by LIPIDMAPS. a) DIUTHAME ion adducts. b) MALDI ion adducts. c) DIUTHAME lipid classes. d) MALDI lipid classes. PC = phosphatidylcholine, PE = phosphatidylethanolamine, PS = phosphatidylserine, PA = Phosphatidic acid, PG = Phosphatidylglycerol, HexCer = Hexosylceramide, PI-Cer = ceramide phosphoinositol, other = belonging to any other lipid class than the ones listed.

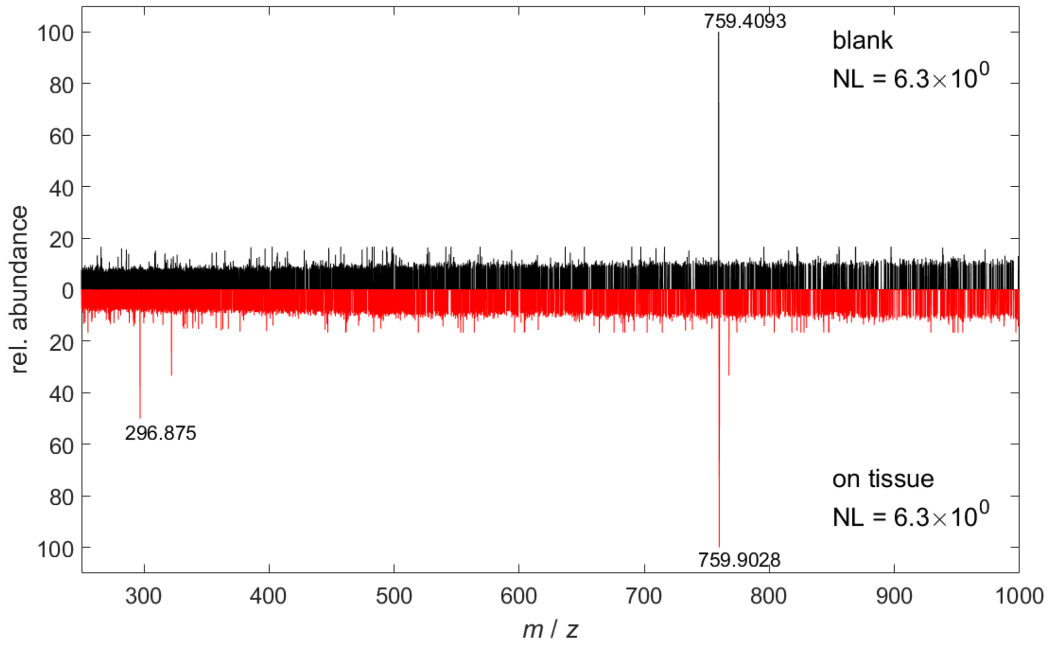

Supporting Figure 5: Comparison of 100 summed-up mass spectra in negative-ion mode acquired using a blank DIUTHAME membrane (black) and a DIUTHAME membrane attached to mouse kidney tissue (red).

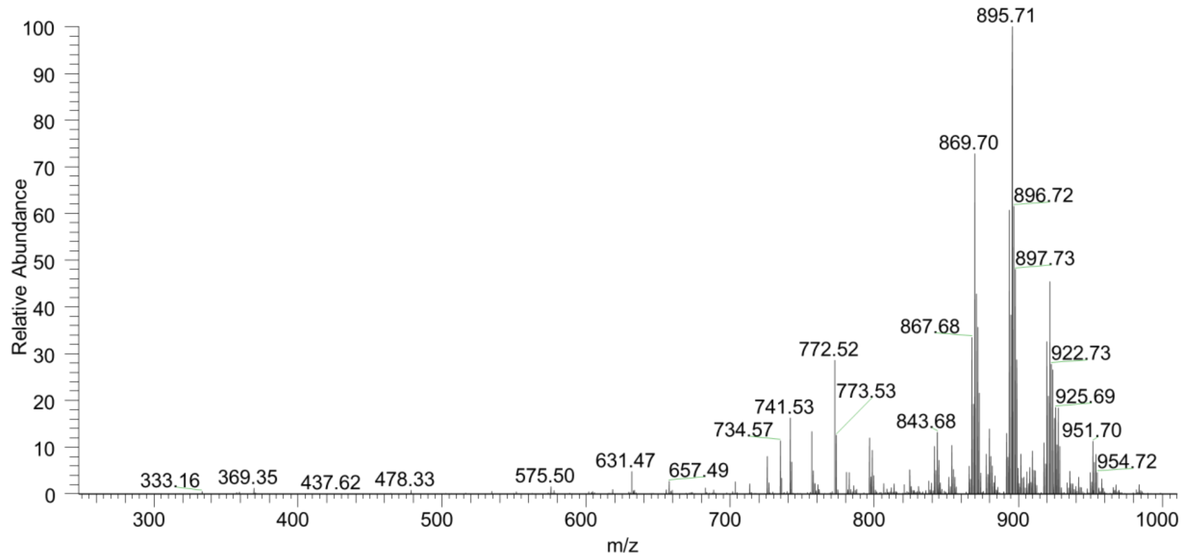

Supporting Figure 6: 100 summed-up mass spectra from mouse kidney tissue investigated by DIUTHAME MSI in positive ion mode showing no significant signal in the lower mass range below m/z 500. Normalized level: 1.37·10³.

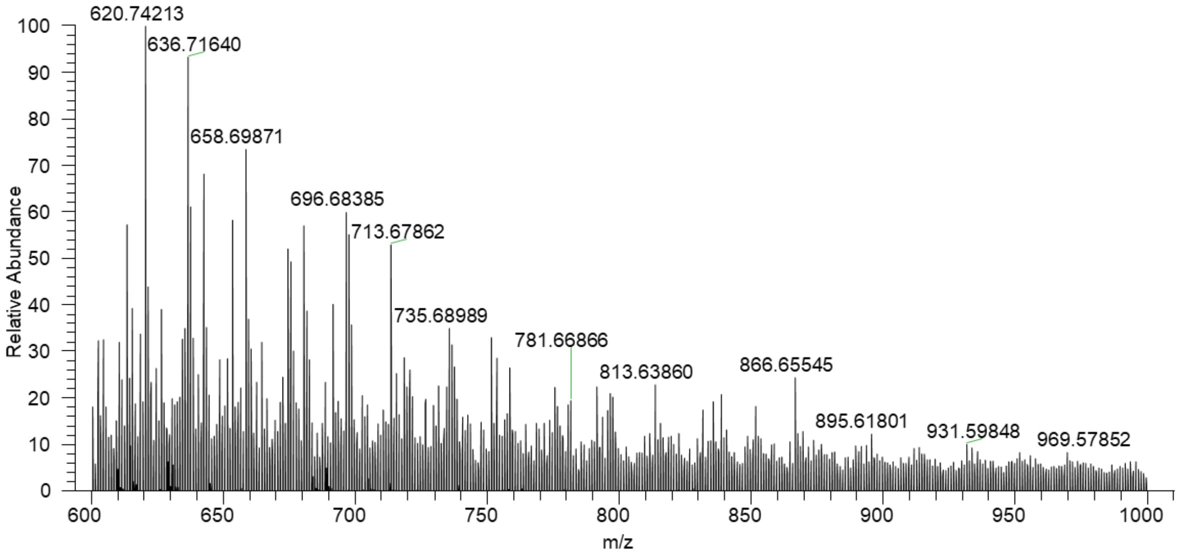

Supporting Figure 7: Single LDI mass spectrum from mouse brain cerebellum. All parameters were the same as for the imaging experiment shown in Figure 2e.

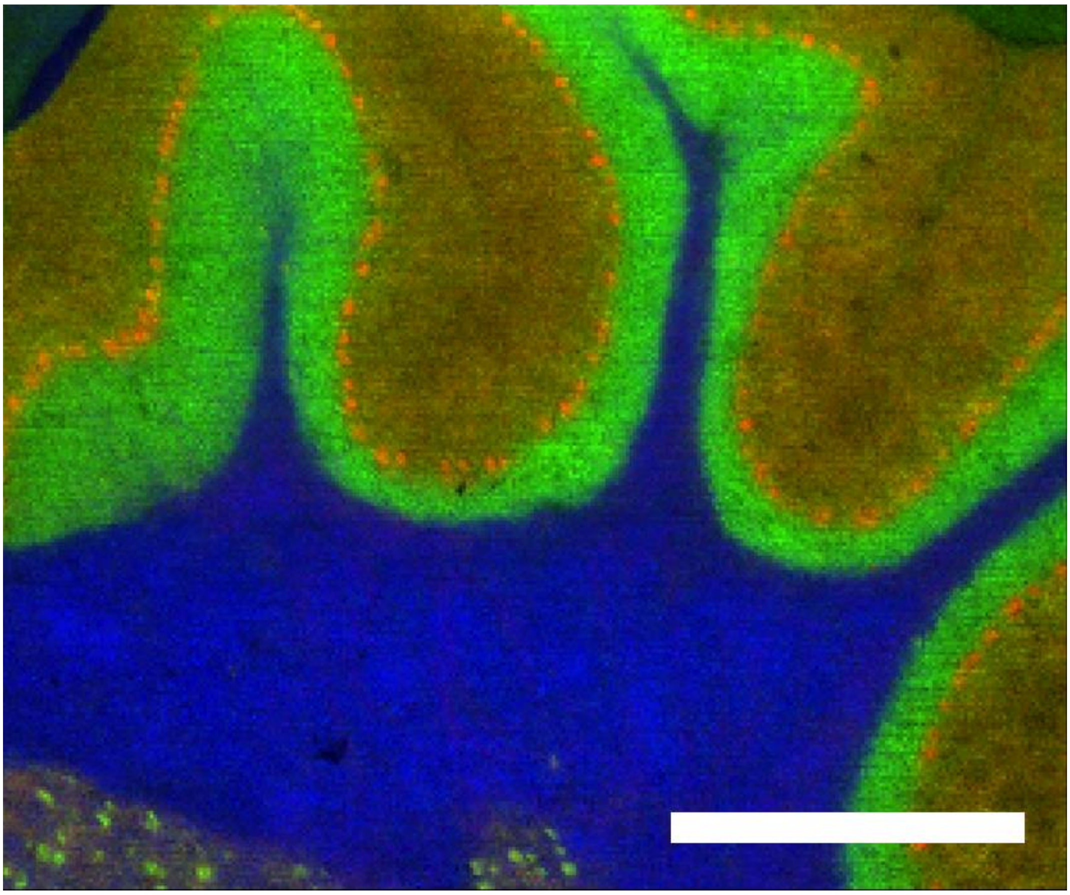

Supporting Figure 8: MALDI MS image of mouse brain cerebellum with 300x250 pixels and 5 µm pixel size. Color coding: red: m/z 872.5525 [SHexCer t38:2 + Na]$^+$, green: m/z 844.5212 [SHexCer t36:2 + Na]$^+$, blue: m/z 848.6329 [PS 40:0 + H]$^+$. Markers of the Purkinje cells (red) where observed in the MALDI experiment, while being absent in the DIUTHAME measurements. Scale bar: 500 µm.

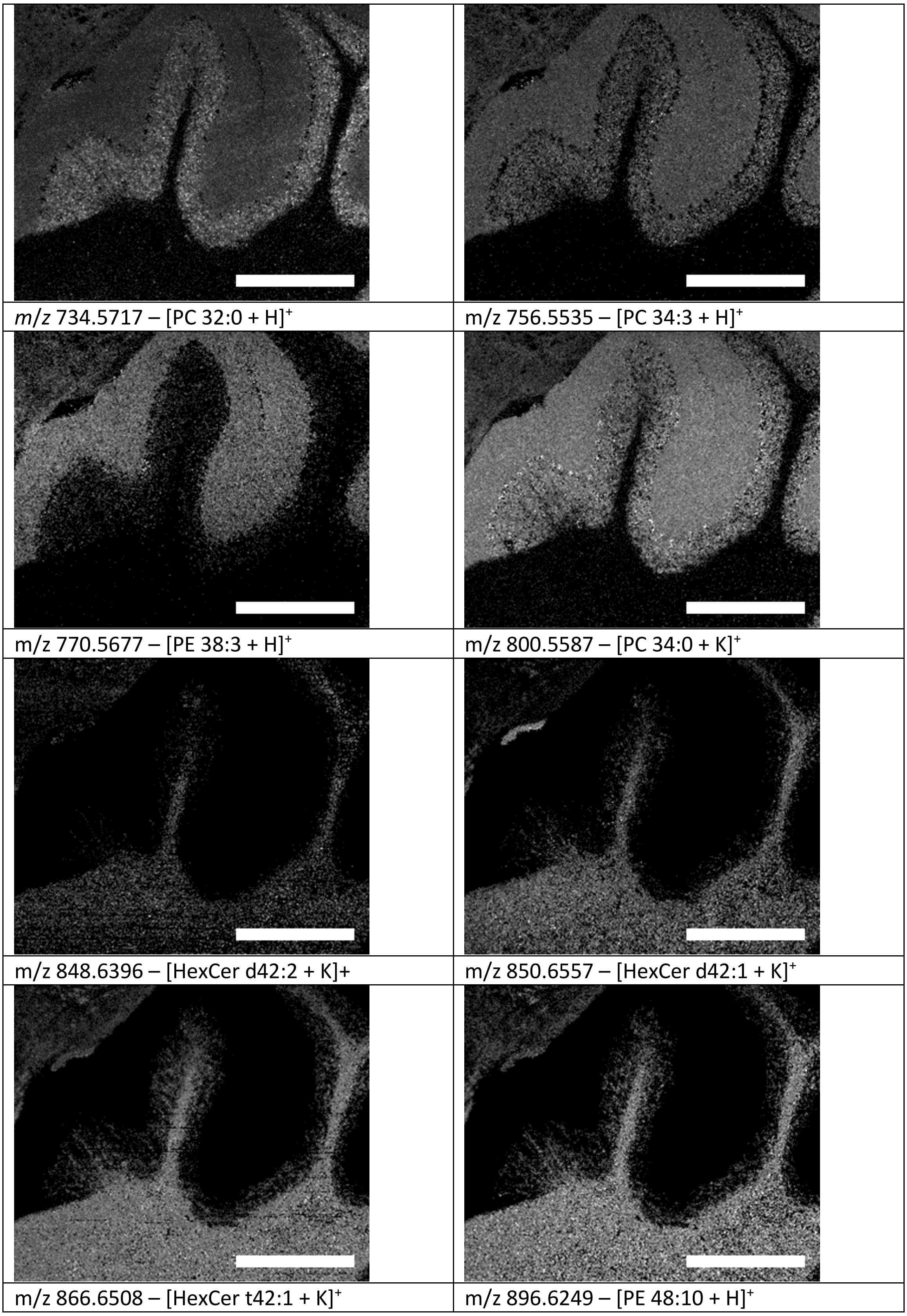

Supporting Figure 9: DIUTHAME MSI of a mouse brain cerebellum section in positive-ion mode. Pixel size: 5 µm. Image size: 300x250 pixels. Scale bars: 500 µm.

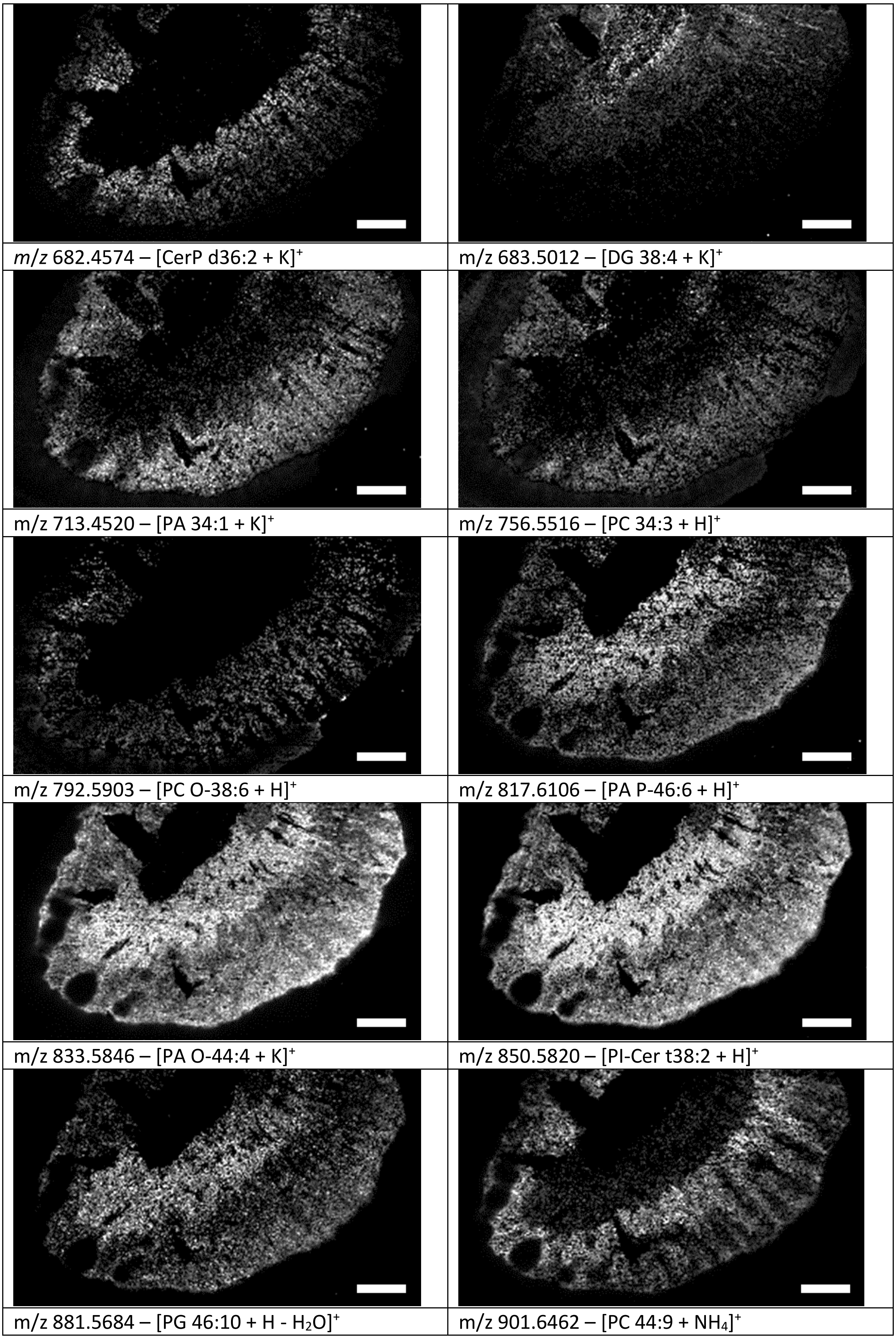

Supporting Figure 10: DIUTHAME MSI of a mouse kidney section in positive-ion mode. Pixel size: 30 µm. Image size: 276x161 pixels. Scale bar: 1 mm.

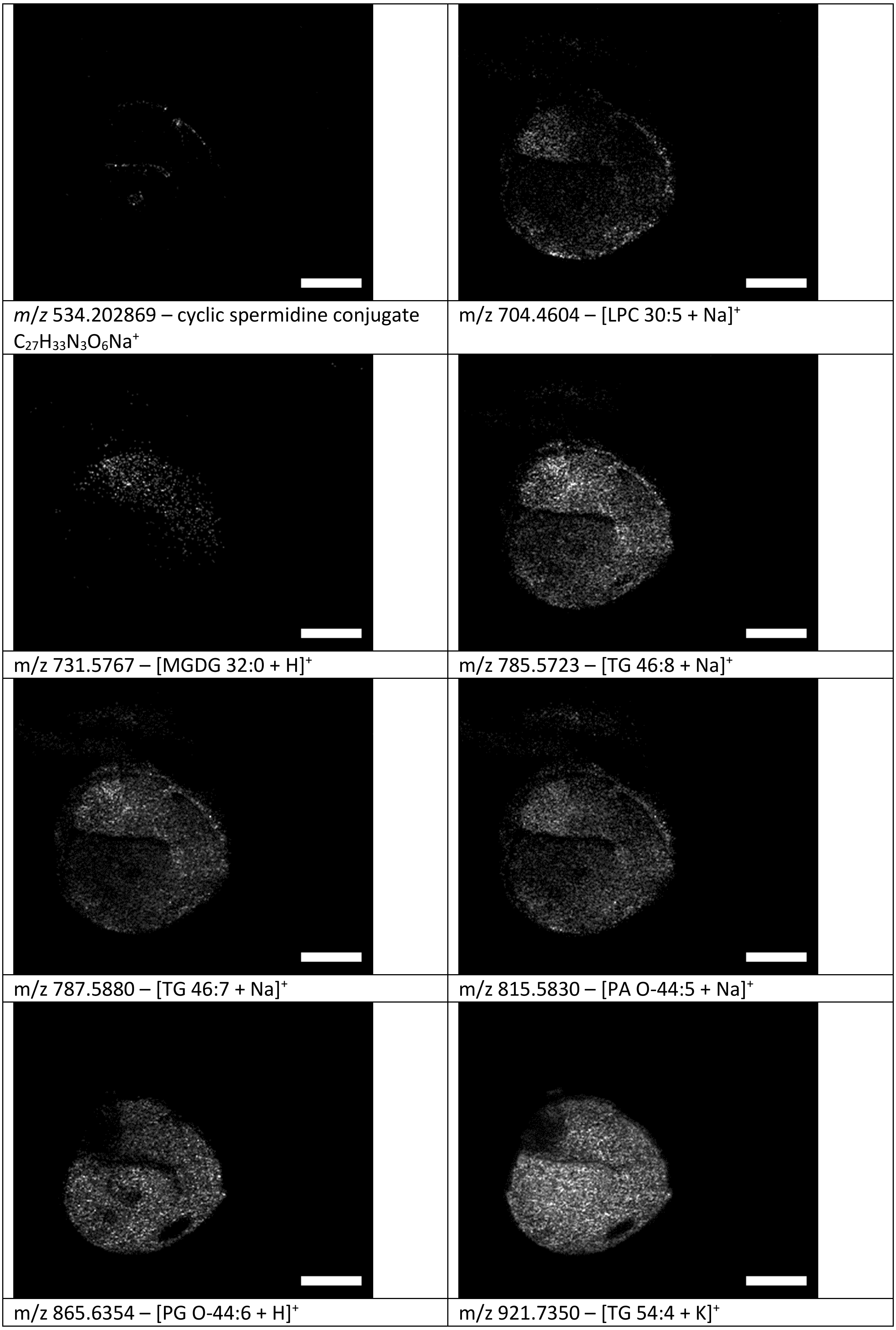

Supporting Figure 11: DIUTHAME MSI of a germinating rapeseed section in positive-ion mode. Pixel size: 20 µm. Image size: 297x245 pixels. Scale bar: 1 mm.

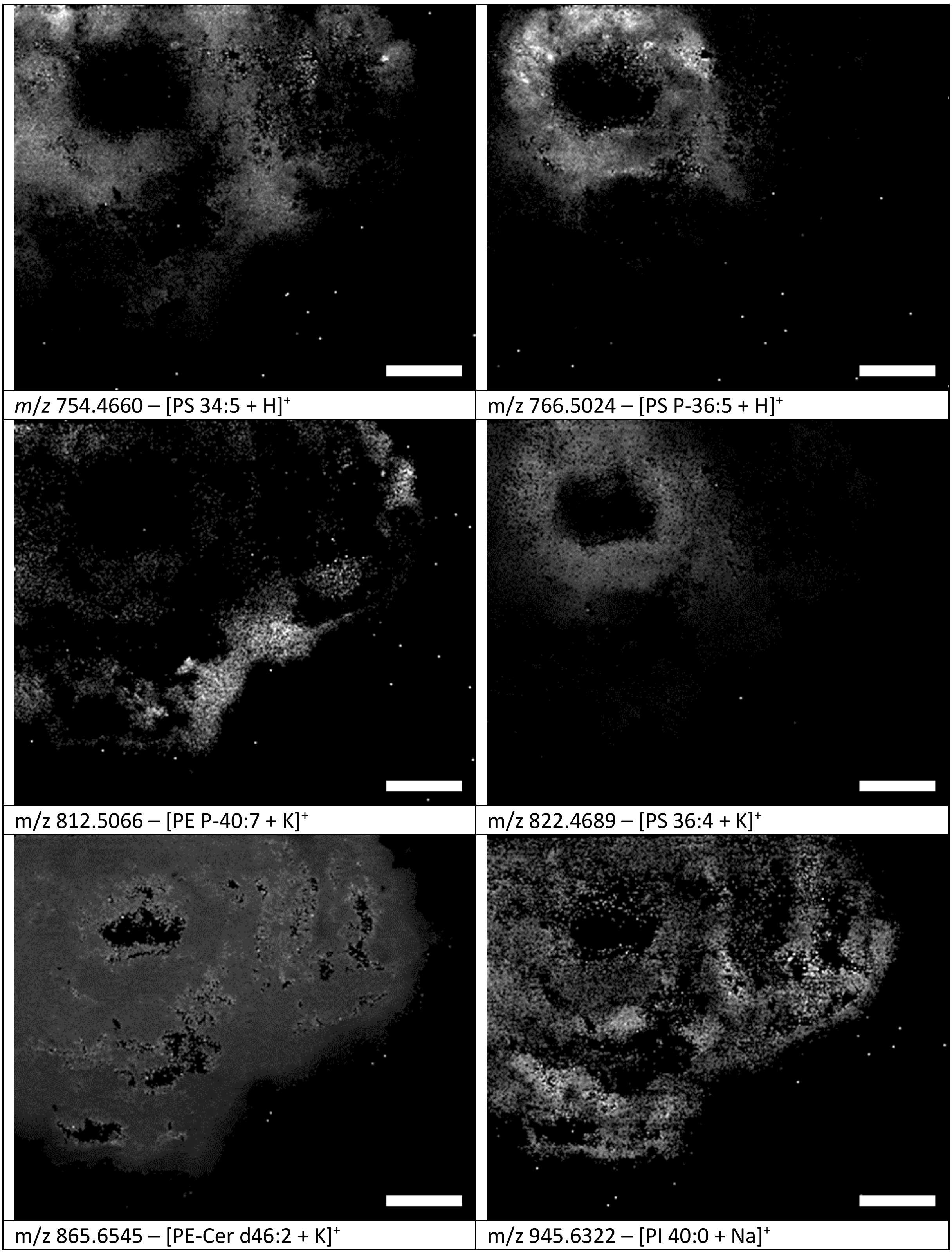

Supporting Figure 12: DIUTHAME MSI of a Spodoptera littoralis larva section in positive-ion mode. Pixel size: 20 µm. Image size: 300x250 pixels. Scale bar: 1mm.

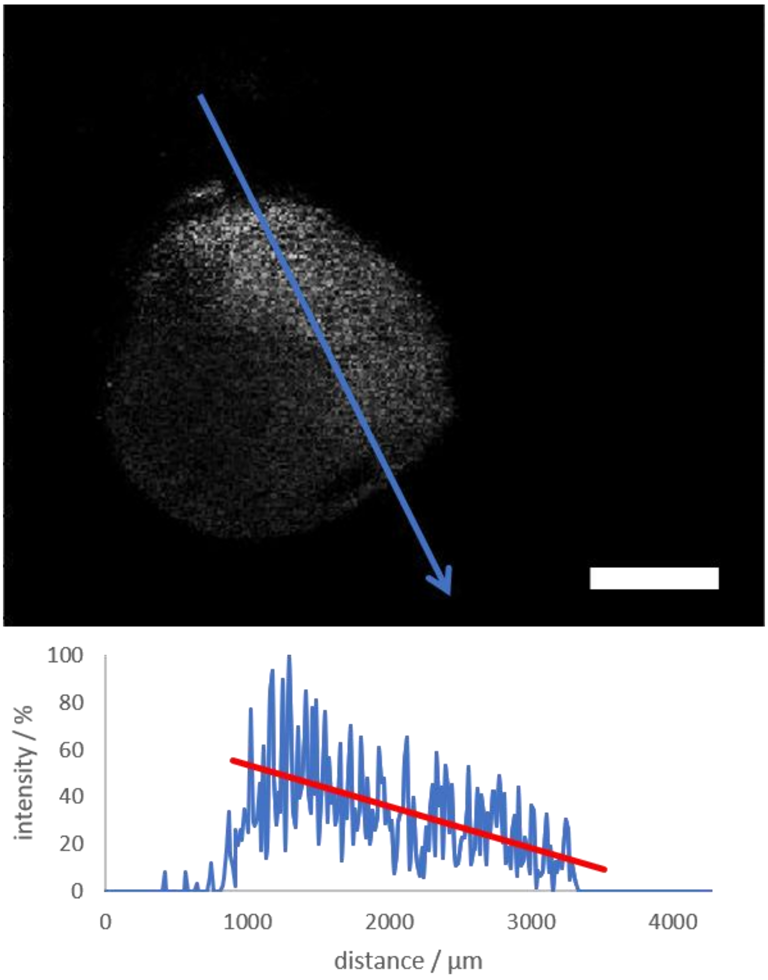

Supporting Figure 13: Line scan through a part of the rapeseed sample measured by DIUTHAME and shown in Figure 3 for m/z 909.6985 [LacCer d36:0 + NH_4]$^+$, indicating a decrease of the compound from the root tip towards the endosperm. Linear regression for the linear part from 1100 µm – 3320 µm is overlaid. Direction and position of the line scan indicated by blue arrow. Scale bar: 1 mm.

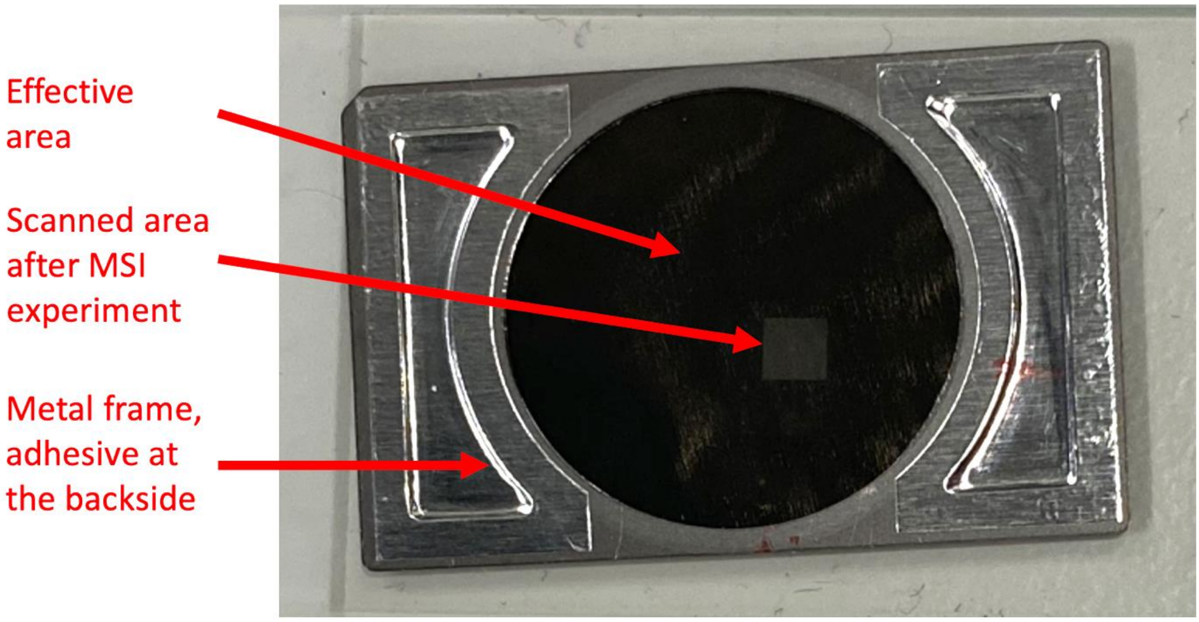

Supporting Figure 14: Optical image of the DIUTHAME membrane attached to a sample on a glass slide. The sample cannot be seen by eye, only under a microscope with transmitted light setting.

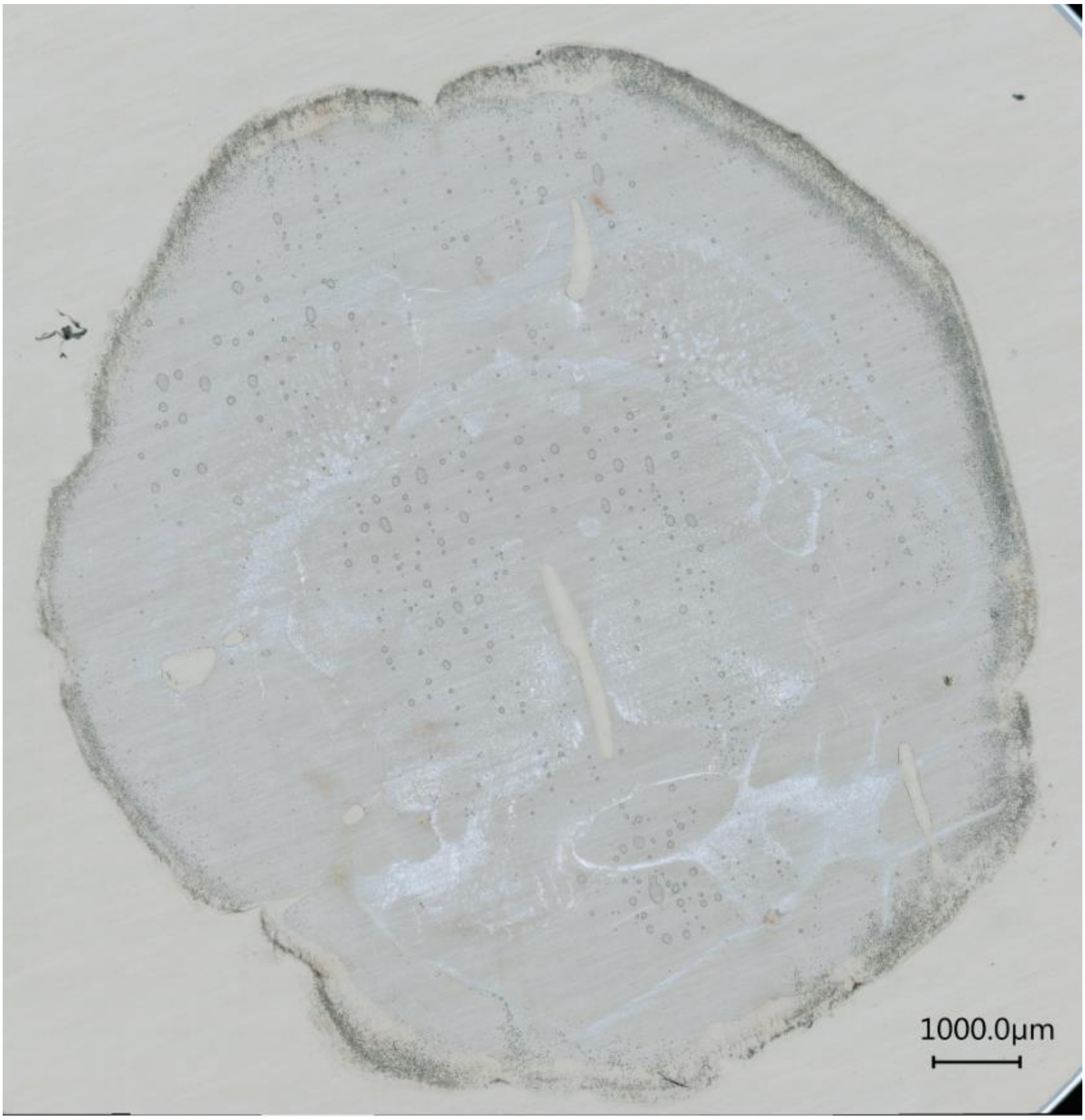

Supporting Figure 15: Transmission light optical microscopy image of a horizontal mouse brain section with DIUTHAME membrane completely attached on top of the sample.

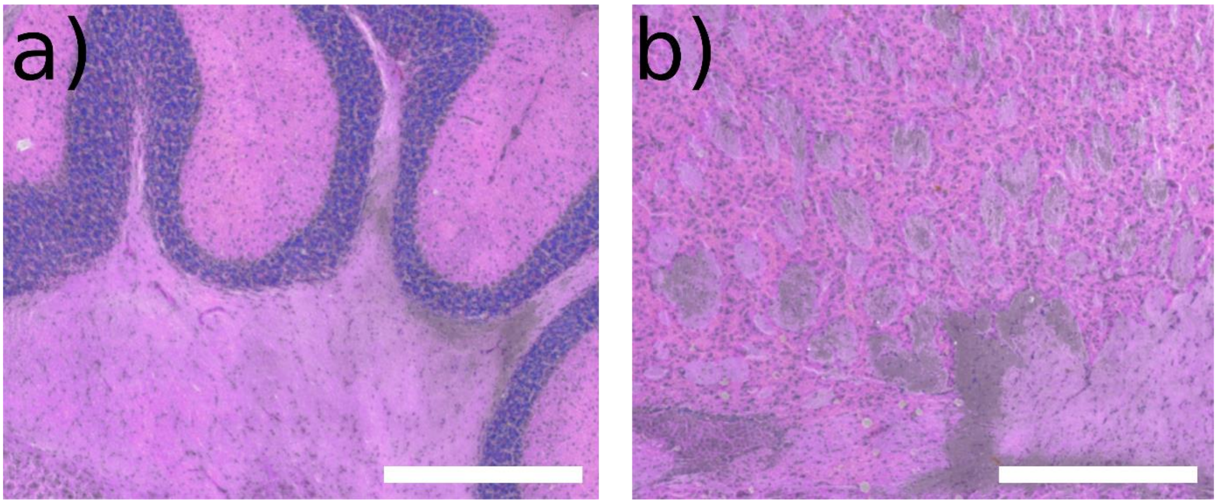

Supporting Figure 16: Optical microscopy image of regions of a mouse brain section stained with hematoxylin and eosin after the MALDI MSI experiment shown in Figure 2. a) Region in cerebellum. b) Striatum ventral region. Scale bars: 500 µm.

Supporting Table 1: List of mass signals detected by DIUTHAME MS from mouse brain cerebellum and annotated by LIPIDMAPS in shorthand notation [1,2].

Number	m/z_{measured}	$m/z_{\text{literature}}$	$\Delta_{m/z}$ / *ppm*	annotated lipid	ion adduct
1	620.4278	620.4286	1.3	PC(24:1)	$[M+H]^+$
2	650.4385	650.4391	0.9	PE(28:1(OH))	$[M+H]^+$
3	660.3995	660.4000	0.8	LPE(30:6)	$[M+Na]^+$
4	672.4205	672.4211	0.9	PE(28:1(OH))	$[M+Na]^+$
5	678.4698	678.4704	0.9	PE(30:1(OH))	$[M+H]^+$
6	686.4152	686.4158	0.9	PC(26:1)	$[M+K]^+$
7	687.4356	687.4362	0.9	PA(32:0)	$[M+K]^+$
8	688.4308	688.4313	0.7	LPE(32:6)	$[M+Na]^+$
9	697.4773	697.4779	0.9	PA(34:1)	$[M+Na]^+$
10	700.4518	700.4524	0.9	PE(30:1(OH))	$[M+Na]^+$
11	702.4101	702.4105	0.6	PE(32:6)	$[M+Na]^+$
12	710.4881	710.4885	0.6	CerP(d38:2)	$[M+K]^+$
13	711.4357	711.4362	0.7	PA(34:2)	$[M+K]^+$
14	713.4515	713.4518	0.4	PA(34:1)	$[M+K]^+$
15	714.4548	714.4552	0.6	PI-Cer(t28:0)	$[M+H]^+$
16	716.4258	716.4263	0.7	PE(30:1(OH))	$[M+K]^+$
17	731.6058	731.6061	0.4	SM(d36:1)	$[M+H]^+$
18	732.4208	732.4211	0.4	LPS(32:6)	$[M+Na]^+$
19	733.4242	733.4262	2.7	LPI(26:1)	$[M+Na]^+$
20	734.5692	734.5694	0.3	PC(32:0)	$[M+H]^+$
21	739.4673	739.4675	0.3	PA(36:2)	$[M+K]^+$
22	740.4706	740.4708	0.3	PI-Cer(t30:1)	$[M+H]^+$
23	741.4828	741.4831	0.4	PA(36:1)	$[M+K]^+$
24	742.4862	742.4865	0.4	PI-Cer(t30:0)	$[M+H]^+$
25	744.4572	744.4575	0.4	PC(32:6)	$[M+Na]^+$
26	748.5846	748.5851	0.7	PE(36:0)	$[M+H]^+$
27	753.5877	753.5881	0.5	SM(d36:1)	$[M+Na]^+$
28	756.4937	756.494	0.4	PE(34:1)	$[M+K]^+$
29	756.5510	756.5514	0.5	PC(32:0)	$[M+Na]^+$
30	758.5093	758.5095	0.3	LPC(34:6)	$[M+Na]^+$
31	760.5849	760.5851	0.3	PC(34:1)	$[M+H]^+$
32	762.6003	762.6007	0.5	PC(34:0)	$[M+H]^+$
33	766.5590	766.5593	0.4	PS(O-34:0(OH))	$[M+H]^+$
34	767.4985	767.4988	0.4	PA(38:2)	$[M+K]^+$
35	769.5619	769.5620	0.1	SM(d36:1)	$[M+K]^+$
36	770.5094	770.5095	0.1	PE(P-38:6)	$[M+Na]^+$
37	770.5652	770.5670	2.3	PE(36:0)	$[M+Na]^+$
38	772.5251	772.5252	0.1	PE(O-38:6)	$[M+Na]^+$
39	773.5285	773.5303	2.3	PG(34:0)	$[M+Na]^+$
40	775.5265	775.5272	0.9	PA(42:7)	$[M+H]^+$
41	782.5095	782.5097	0.3	PE(36:2)	$[M+K]^+$
42	782.5541	782.5542	0.1	PI-Cer(d34:0)	$[M+H]^+$

43	782.5670	782.5670	< 0.1	PC(34:1)	$[M+Na]^+$
44	784.5251	784.5253	0.3	PE(36:1)	$[M+K]^+$
45	784.5824	784.5827	0.4	PC(34:0)	$[M+Na]^+$
46	786.5407	786.5408	0.1	PC(P-36:5)	$[M+Na]^+$
47	788.6163	788.6164	0.1	PC(36:1)	$[M+H]^+$
48	796.5252	796.5252	< 0.1	PE(P-40:7)	$[M+Na]^+$
49	797.5932	797.5933	0.1	SM(d38:1)	$[M+K]^+$
50	798.5410	798.5410	< 0.1	PC(34:1)	$[M+K]^+$
51	799.5444	799.5460	2.0	PG(36:1)	$[M+Na]^+$
52	800.5566	800.5566	< 0.1	PC(34:0)	$[M+K]^+$
53	801.5599	801.5616	2.1	PG(36:0)	$[M+Na]^+$
54	806.5090	806.5097	0.9	PE(38:4)	$[M+K]^+$
55	808.5696	808.5698	0.2	PI-Cer(d36:1)	$[M+H]^+$
56	810.5855	810.5855	< 0.1	PI-Cer(d36:0)	$[M+H]^+$
57	810.5985	810.5983	0.2	PC(36:1)	$[M+Na]^+$
58	814.5358	814.5359	0.1	PC(34:1(OH))	$[M+K]^+$
59	822.6218	822.6219	0.1	PS(O-38:0(OH))	$[M+H]^+$
60	822.6427	822.6429	0.2	HexCer(t40:1)	$[M+Na]^+$
61	824.5566	824.5566	< 0.1	PC(36:2)	$[M+K]^+$
62	826.5724	826.5723	0.1	PC(36:1)	$[M+K]^+$
63	827.5757	827.5773	1.9	PG(38:1)	$[M+Na]^+$
64	828.5151	828.5151	< 0.1	PS(36:1)	$[M+K]^+$
65	830.5097	830.5097	< 0.1	PE(40:6)	$[M+K]^+$
66	832.5100	832.5099	0.1	PS(38:5)	$[M+Na]^+$
67	836.6009	836.6011	0.2	PI-Cer(d38:1)	$[M+H]^+$
68	838.6168	838.6168	< 0.1	PI-Cer(d38:0)	$[M+H]^+$
69	840.6230	840.6243	1.5	CerP(t46:1)	$[M+K]^+$
70	840.6325	840.6325	< 0.1	HexCer(t40:0)	$[M+K]^+$
71	846.5254	846.5257	0.4	PI-Cer(d36:1)	$[M+K]^+$
72	848.6375	848.6375	< 0.1	PS(40:0)	$[M+H]^+$
73	850.6532	850.6532	< 0.1	PS(O-40:0(OH))	$[M+H]^+$
74	850.6739	850.6742	0.4	HexCer(t42:1)	$[M+Na]^+$
75	864.6324	864.6324	< 0.1	PI-Cer(d40:1)	$[M+H]^+$
76	866.6483	866.6482	0.1	HexCer(t42:1)	$[M+K]^+$
77	868.6546	868.6556	1.2	CerP(t48:1)	$[M+K]^+$

Supporting Table 2: List of mass signals detected by MALDI MS from mouse brain cerebellum and annotated by LIPIDMAPS in shorthand notation [1,2].

Number	m/z measured	m/z literature	$\Delta_{m/z}$ / ppm	annotated lipid	ion adduct
1	606.4141	606.4129	2.0	PE(26:1)	$[M+H]^+$
2	620.4295	620.4286	1.5	PC(24:1)	$[M+H]^+$
3	622.4451	622.4442	1.4	PC(24:0)	$[M+H]^+$
4	633.4868	633.4855	2.1	DG(34:1)	$[M+K]^+$
5	634.4451	634.4442	1.4	PE(28:1)	$[M+H]^+$
6	642.4113	642.4105	1.2	PC(24:1)	$[M+Na]^+$
7	644.4271	644.4262	1.4	PC(24:0)	$[M+Na]^+$
8	648.4607	648.4599	1.2	PC(26:1)	$[M+H]^+$
9	650.4399	650.4391	1.2	PE(28:1(OH))	$[M+H]^+$
10	650.4762	650.4755	1.1	PC(26:0)	$[M+H]^+$
11	652.4192	652.4185	1.1	HexCer(d28:2)	$[M+K]^+$
12	656.4269	656.4262	1.1	PE(28:1)	$[M+Na]^+$
13	661.4042	661.4051	1.4	PG(26:0)	$[M+Na]^+$
14	661.5176	661.5168	1.2	DG(36:1)	$[M+K]^+$
15	664.4555	664.4548	1.1	LPS(28:1)	$[M+H]^+$
16	667.4381	667.4392	1.6	MGDG(26:1)	$[M+Na]^+$
17	668.4140	668.4133	1.0	PI-Cer(d26:1)	$[M+H]^+$
18	670.4425	670.4418	1.0	PC(26:1)	$[M+Na]^+$
19	672.4218	672.4211	1.0	PE(28:1(OH))	$[M+Na]^+$
20	672.4582	672.4575	1.0	PC(26:0)	$[M+Na]^+$
21	674.4160	674.4158	0.3	PE(28:0)	$[M+K]^+$
22	678.4708	678.4704	0.6	PE(30:1(OH))	$[M+H]^+$
23	680.4502	680.4498	0.6	HexCer(d30:2)	$[M+K]^+$
24	682.4580	682.4572	1.2	CerP(d36:2)	$[M+K]^+$
25	683.5022	683.5011	1.6	DG(38:4)	$[M+K]^+$
26	684.4738	684.4729	1.3	CerP(d36:1)	$[M+K]^+$
27	685.4048	685.4051	0.4	PG(28:2)	$[M+Na]^+$
28	685.4215	685.4205	1.5	PA(32:1)	$[M+K]^+$
29	686.4161	686.4158	0.4	PC(26:1)	$[M+K]^+$
30	687.4200	687.4208	1.2	PG(28:1)	$[M+Na]^+$
31	687.4371	687.4362	1.3	PA(32:0)	$[M+K]^+$
32	688.4314	688.4314	< 0.1	PC(26:0)	$[M+K]^+$
33	689.4197	689.4177	2.9	PA(36:8)	$[M+H]^+$
34	689.4349	689.4364	2.2	PG(28:0)	$[M+Na]^+$
35	692.4864	692.4861	0.4	LPS(30:1)	$[M+H]^+$
36	693.4456	693.4466	1.4	PA(34:3)	$[M+Na]^+$
37	694.5155	694.5146	1.3	CerP(d38:2)	$[M+Na]^+$
38	695.4422	695.4412	1.4	PA(O-34:3)	$[M+K]^+$
39	695.4687	695.4705	2.6	MGDG(28:1)	$[M+Na]^+$
40	696.4449	696.4447	0.3	HexCer(t30:2)	$[M+K]^+$
41	697.4789	697.4779	1.4	PA(34:1)	$[M+Na]^+$
42	698.4163	698.4158	0.7	PE(30:2)	$[M+K]^+$

43	699.4853	699.4838	2.1	PE-Cer(d34:1)	$[M+K]^+$
44	700.4319	700.4314	0.7	PE(30:1)	$[M+K]^+$
45	700.4529	700.4524	0.7	PE(30:1(OH))	$[M+Na]^+$
46	701.4549	701.4542	1.0	DG(40:9)	$[M+K]^+$
47	702.4110	702.4107	0.4	LPS(28:1)	$[M+K]^+$
48	703.4144	703.4157	1.8	PG(28:1(OH))	$[M+Na]^+$
49	706.5386	706.5381	0.7	PC(30:0)	$[M+H]^+$
50	707.5021	707.5011	1.4	DG(40:6)	$[M+K]^+$
51	708.4813	708.4811	0.3	HexCer(d32:2)	$[M+K]^+$
52	709.4192	709.4191	0.1	SQDG(26:1)	$[M+H]^+$
53	710.4744	710.4755	1.5	PE(34:5)	$[M+H]^+$
54	710.4892	710.4885	1.0	CerP(d38:2)	$[M+K]^+$
55	711.4197	711.4208	1.5	PG(30:3)	$[M+Na]^+$
56	711.4372	711.4362	1.4	PA(34:2)	$[M+K]^+$
57	712.4317	712.4314	0.4	PC(28:2)	$[M+K]^+$
58	712.4410	712.4395	2.1	PI-Cer(t28:1)	$[M+H]^+$
59	712.4877	712.4888	1.5	PE(32:1)	$[M+Na]^+$
60	713.4367	713.4364	0.4	PG(30:2)	$[M+Na]^+$
61	713.4525	713.4518	1.0	PA(34:1)	$[M+K]^+$
62	714.4111	714.4107	0.6	PE(30:2(OH))	$[M+K]^+$
63	714.4563	714.4552	1.5	PI-Cer(t28:0)	$[M+H]^+$
64	715.4505	715.4521	2.2	PG(30:1)	$[M+Na]^+$
65	716.4267	716.4263	0.6	PE(30:1(OH))	$[M+K]^+$
66	716.4622	716.4626	0.6	LPE(34:6)	$[M+Na]^+$
67	717.4505	717.4490	2.1	PA(38:8)	$[M+H]^+$
68	718.4061	718.4056	0.7	PS(28:0)	$[M+K]^+$
69	718.4245	718.4266	2.9	PI-Cer(d28:1)	$[M+Na]^+$
70	719.4609	719.4622	1.8	PA(36:4)	$[M+Na]^+$
71	720.5546	720.5538	1.1	PE(34:0)	$[M+H]^+$
72	721.4781	721.4779	0.3	PA(36:3)	$[M+Na]^+$
73	723.4744	723.4725	2.6	PA(O-36:3)	$[M+K]^+$
74	723.4942	723.4935	1.0	PA(36:2)	$[M+Na]^+$
75	724.4762	724.4760	0.3	HexCer(t32:2)	$[M+K]^+$
76	725.4884	725.4882	0.3	PA(O-36:2)	$[M+K]^+$
77	725.5105	725.5116	1.5	PA(38:4)	$[M+H]^+$
78	725.5577	725.5568	1.2	SM(d34:1)	$[M+Na]^+$
79	726.4110	726.4107	0.4	LPS(30:3)	$[M+K]^+$
80	726.4476	726.4471	0.7	PE(32:2)	$[M+K]^+$
81	726.4916	726.4916	< 0.1	PI-Cer(d30:0)	$[M+H]^+$
82	727.4683	727.4673	1.4	PA(P-38:6)	$[M+Na]^+$
83	728.4266	728.4263	0.4	LPS(30:2)	$[M+K]^+$
84	728.4629	728.4627	0.3	PE(32:1)	$[M+K]^+$
85	728.4844	728.4837	1.0	PE(32:1(OH))	$[M+Na]^+$
86	728.5207	728.5201	0.8	PC(30:0)	$[M+Na]^+$
87	730.5749	730.5745	0.5	PE(O-36:2)	$[M+H]^+$

88	731.6068	731.6061	1.0	SM(d36:1)	$[M+H]^+$
89	732.4215	732.4211	0.5	LPS(32:6)	$[M+Na]^+$
90	732.5545	732.5538	1.0	PC(32:1)	$[M+H]^+$
91	733.4245	733.4262	2.3	LPI(26:1)	$[M+Na]^+$
92	734.4199	734.4215	2.2	PI-Cer(t28:1)	$[M+Na]^+$
93	734.5701	734.5694	1.0	PC(32:0)	$[M+H]^+$
94	735.4345	735.4348	0.4	SQDG(28:2)	$[M+H]^+$
95	736.4377	736.4371	0.8	PI-Cer(t28:0)	$[M+Na]^+$
96	737.4521	737.4518	0.4	PA(36:3)	$[M+K]^+$
97	738.4114	738.4107	0.9	PE(32:4(OH))	$[M+K]^+$
98	738.4551	738.4552	0.1	PI-Cer(t30:2)	$[M+H]^+$
99	738.5205	738.5198	0.9	CerP(d40:2)	$[M+K]^+$
100	739.4510	739.4521	1.5	PG(32:3)	$[M+Na]^+$
101	739.4680	739.4675	0.7	PA(36:2)	$[M+K]^+$
102	740.4119	740.4109	1.4	PS(30:3(OH))	$[M+Na]^+$
103	740.4717	740.4708	1.2	PI-Cer(t30:1)	$[M+H]^+$
104	740.4986	740.4991	0.7	PE(O-34:2)	$[M+K]^+$
105	740.5203	740.5201	0.3	PE(34:1)	$[M+Na]^+$
106	741.4487	741.4490	0.4	PA(40:10)	$[M+H]^+$
107	741.4676	741.4677	0.1	PG(32:2)	$[M+Na]^+$
108	741.4846	741.4831	2.0	PA(36:1)	$[M+K]^+$
109	741.5311	741.5307	0.5	SM(d34:1)	$[M+K]^+$
110	742.4065	742.4056	1.2	PS(30:2)	$[M+K]^+$
111	742.4424	742.4420	0.5	PE(32:2(OH))	$[M+K]^+$
112	742.4875	742.4865	1.3	PI-Cer(t30:0)	$[M+H]^+$
113	742.5155	742.5147	1.1	PE(O-34:1)	$[M+K]^+$
114	742.5351	742.5357	0.8	PE(34:0)	$[M+Na]^+$
115	743.4818	743.4834	2.2	PG(32:1)	$[M+Na]^+$
116	744.4578	744.4576	0.3	PE(32:1(OH))	$[M+K]^+$
117	744.4946	744.4940	0.8	PC(30:0)	$[M+K]^+$
118	744.5540	744.5538	0.3	PE(36:2)	$[M+H]^+$
119	745.4778	745.4779	0.1	PA(38:5)	$[M+Na]^+$
120	745.4978	745.4990	1.6	PG(32:0)	$[M+Na]^+$
121	745.6227	745.6218	1.2	PE-Cer(d40:1)	$[M+H]^+$
122	746.5701	746.5694	0.9	PE(36:1)	$[M+H]^+$
123	746.6061	746.6058	0.4	CerP(t42:1)	$[M+H]^+$
124	747.4714	747.4725	1.5	PA(O-38:5)	$[M+K]^+$
125	747.4929	747.4935	0.8	PA(38:4)	$[M+Na]^+$
126	748.5858	748.5851	0.9	PE(36:0)	$[M+H]^+$
127	749.5100	749.5092	1.1	PA(38:3)	$[M+Na]^+$
128	750.5421	750.5432	1.5	PE(O-38:6)	$[M+H]^+$
129	750.5856	750.5854	0.3	DGCC(32:0)	$[M+Na]^+$
130	751.5252	751.5248	0.5	PA(38:2)	$[M+Na]^+$
131	751.5731	751.5724	0.9	SM(d36:2)	$[M+Na]^+$
132	752.4116	752.4111	0.7	PI-Cer(t28:0)	$[M+K]^+$

133	752.4271	752.4263	1.1	LPS(32:4)	[M+K]$^+$
134	752.5074	752.5073	0.1	HexCer(t34:2)	[M+K]$^+$
135	752.5571	752.5565	0.8	PE(O-36:2)	[M+Na]$^+$
136	753.4835	753.4855	2.7	DG(44:11)	[M+K]$^+$
137	753.5886	753.5881	0.7	SM(d36:1)	[M+Na]$^+$
138	754.4788	754.4784	0.5	PE(34:2)	[M+K]$^+$
139	754.5364	754.5357	0.9	PC(32:1)	[M+Na]$^+$
140	755.4070	755.4049	2.8	PA(38:8)	[M+K]$^+$
141	755.4630	755.4624	0.8	PA(36:2(OH))	[M+K]$^+$
142	755.4996	755.4986	1.3	PA(P-40:6)	[M+Na]$^+$
143	755.5446	755.5464	2.4	PE-Cer(d38:1)	[M+K]$^+$
144	756.4944	756.4940	0.5	PE(34:1)	[M+K]$^+$
145	756.5519	756.5514	0.7	PC(32:0)	[M+Na]$^+$
146	757.4192	757.4191	0.1	SQDG(30:5)	[M+H]$^+$
147	758.4929	758.4943	1.8	PS(32:0)	[M+Na]$^+$
148	758.5104	758.5097	0.9	PE(34:0)	[M+K]$^+$
149	758.5711	758.5694	2.2	PC(34:2)	[M+H]$^+$
150	759.4360	759.4362	0.3	PA(38:6)	[M+K]$^+$
151	759.6383	759.6374	1.2	SM(d38:1)	[M+H]$^+$
152	760.4389	760.4371	2.4	PI-Cer(t30:2)	[M+Na]$^+$
153	760.4526	760.4524	0.3	LPS(34:6)	[M+Na]$^+$
154	760.5854	760.5851	0.4	PC(34:1)	[M+H]$^+$
155	761.4507	761.4504	0.4	SQDG(30:3)	[M+H]$^+$
156	762.4541	762.4528	1.7	PI-Cer(t30:1)	[M+Na]$^+$
157	762.4817	762.4834	2.2	PE(O-36:5)	[M+K]$^+$
158	762.6026	762.6007	2.5	PC(34:0)	[M+H]$^+$
159	763.4663	763.4661	0.3	SQDG(30:2)	[M+H]$^+$
160	764.4695	764.4684	1.4	PI-Cer(t30:0)	[M+Na]$^+$
161	764.4986	764.4991	0.7	PE(O-36:4)	[M+K]$^+$
162	765.4661	765.4677	2.1	PG(34:4)	[M+Na]$^+$
163	765.4840	765.4831	1.2	PA(38:3)	[M+K]$^+$
164	766.4274	766.4266	1.0	PS(32:4(OH))	[M+Na]$^+$
165	766.4870	766.4865	0.7	PI-Cer(t32:2)	[M+H]$^+$
166	766.5147	766.5147	< 0.1	PE(O-36:3)	[M+K]$^+$
167	766.5359	766.5357	0.3	PE(36:2)	[M+Na]$^+$
168	766.5592	766.5593	0.1	PS(O-34:0(OH))	[M+H]$^+$
169	766.5786	766.5803	2.2	HexCer(t36:1)	[M+Na]$^+$
170	767.4820	767.4834	1.8	PG(34:3)	[M+Na]$^+$
171	767.4993	767.4988	0.7	PA(38:2)	[M+K]$^+$
172	767.5178	767.5197	2.5	PA(38:2(OH))	[M+Na]$^+$
173	767.5468	767.5464	0.5	SM(d36:2)	[M+K]$^+$
174	768.5030	768.5021	1.2	PI-Cer(t32:1)	[M+H]$^+$
175	768.5306	768.5304	0.3	PE(O-36:2)	[M+K]$^+$
176	768.5515	768.5514	0.1	PE(36:1)	[M+Na]$^+$
177	768.5883	768.5878	0.7	CerP(t42:1)	[M+Na]$^+$

178	769.4784	769.4780	0.5	PG(O-34:3)	$[M+K]^+$
179	769.4985	769.4990	0.6	LBPA(34:2)	$[M+Na]^+$
180	769.5159	769.5144	1.9	PA(38:1)	$[M+K]^+$
181	769.5344	769.5354	1.3	PA(38:1(OH))	$[M+Na]^+$
182	769.5625	769.5620	0.6	SM(d36:1)	$[M+K]^+$
183	770.5103	770.5097	0.8	PC(32:1)	$[M+K]^+$
184	770.5659	770.5670	1.4	PE(36:0)	$[M+Na]^+$
185	771.4951	771.4959	1.0	PA(42:9)	$[M+H]^+$
186	771.5134	771.5147	1.7	LBPA(34:1)	$[M+Na]^+$
187	772.5257	772.5253	0.5	PC(32:0)	$[M+K]^+$
188	772.5634	772.5617	2.2	PE(O-36:0)	$[M+K]^+$
189	772.5840	772.5851	1.4	PE(38:2)	$[M+H]^+$
190	773.5290	773.5303	1.7	PG(34:0)	$[M+Na]^+$
191	774.6010	774.6007	0.4	PE(38:1)	$[M+H]^+$
192	775.4079	775.4063	2.1	SQDG(28:1)	$[M+K]^+$
193	775.5271	775.5272	0.1	PA(42:7)	$[M+H]^+$
194	775.5345	775.5355	1.3	MGDG(36:6)	$[M+H]^+$
195	776.4113	776.4111	0.3	PI-Cer(t30:2)	$[M+K]^+$
196	776.4624	776.4627	0.4	PE(36:5)	$[M+K]^+$
197	776.5596	776.5589	0.9	PE(P-40:6)	$[M+H]^+$
198	776.5927	776.5928	0.1	CerP(d44:3)	$[M+Na]^+$
199	776.6179	776.6164	1.9	PE(38:0)	$[M+H]^+$
200	777.4236	777.4220	2.1	SQDG(28:0)	$[M+K]^+$
201	778.4272	778.4267	0.6	PI-Cer(t30:1)	$[M+K]^+$
202	778.4768	778.4784	2.1	PE(36:4)	$[M+K]^+$
203	779.4799	779.4787	1.5	DGDG(24:1)	$[M+H]^+$
204	780.4424	780.4424	< 0.1	PI-Cer(t30:0)	$[M+K]^+$
205	780.4926	780.4940	1.8	PE(36:3)	$[M+K]^+$
206	780.5521	780.5514	0.9	PC(34:2)	$[M+Na]^+$
207	781.4189	781.4191	0.3	SQDG(32:7)	$[M+H]^+$
208	781.4762	781.4779	2.2	PPA(36:2)	$[M+H]^+$
209	781.4961	781.4944	2.2	DGDG(24:0)	$[M+H]^+$
210	781.6201	781.6194	0.9	SM(d38:1)	$[M+Na]^+$
211	782.5099	782.5097	0.3	PE(36:2)	$[M+K]^+$
212	782.5675	782.5670	0.6	PC(34:1)	$[M+Na]^+$
213	783.4349	783.4348	0.1	SQDG(32:6)	$[M+H]^+$
214	783.5544	783.5534	1.3	PG(O-38:5)	$[M+H]^+$
215	784.5257	784.5253	0.5	PE(36:1)	$[M+K]^+$
216	784.5602	784.5617	1.9	CerP(t42:1)	$[M+K]^+$
217	784.5841	784.5851	1.3	PC(36:3)	$[M+H]^+$
218	785.4516	785.4518	0.3	PA(40:7)	$[M+K]^+$
219	785.5647	785.5667	2.5	PG(O-36:1)	$[M+Na]^+$
220	786.4840	786.4834	0.8	PE(P-38:6)	$[M+K]^+$
221	786.5046	786.5046	< 0.1	PC(32:1(OH))	$[M+K]^+$
222	786.5416	786.5410	0.8	PE(36:0)	$[M+K]^+$

223	786.6014	786.6007	0.9	PC(36:2)	$[M+H]^+$
224	787.4508	787.4521	1.7	PG(36:7)	$[M+Na]^+$
225	787.4680	787.4675	0.6	PA(40:6)	$[M+K]^+$
226	787.4875	787.4884	1.1	PA(40:6(OH))	$[M+Na]^+$
227	787.5077	787.5096	2.4	PG(34:1(OH))	$[M+Na]^+$
228	787.6688	787.6687	0.1	SM(d40:1)	$[M+H]^+$
229	788.4978	788.4991	1.6	PE(O-38:6)	$[M+K]^+$
230	788.5190	788.5201	1.4	PE(38:5)	$[M+Na]^+$
231	788.6165	788.6164	0.1	PC(36:1)	$[M+H]^+$
232	789.4828	789.4831	0.4	PA(40:5)	$[M+K]^+$
233	790.4863	790.4841	2.8	PI-Cer(t32:1)	$[M+Na]^+$
234	790.5139	790.5147	1.0	PE(O-38:5)	$[M+K]^+$
235	790.5356	790.5357	0.1	PE(38:4)	$[M+Na]^+$
236	791.4973	791.4974	0.1	SQDG(32:2)	$[M+H]^+$
237	792.4982	792.4997	1.9	PI-Cer(t32:0)	$[M+Na]^+$
238	792.5298	792.5304	0.8	PE(O-38:4)	$[M+K]^+$
239	792.5540	792.5538	0.3	PE(40:6)	$[M+H]^+$
240	792.5667	792.5668	0.1	CerP(d44:3)	$[M+K]^+$
241	793.4187	793.4181	0.8	PPA(34:1)	$[M+K]^+$
242	793.4762	793.4779	2.1	PA(42:9)	$[M+Na]^+$
243	793.5019	793.5014	0.6	PG(38:7)	$[M+H]^+$
244	793.5144	793.5144	< 0.1	PA(40:3)	$[M+K]^+$
245	793.5575	793.5589	1.8	PG(36:1(OH))	$[M+H]^+$
246	794.5078	794.5083	0.6	SHexCer(t34:2)	$[M+H]^+$
247	794.5174	794.5178	0.5	PI-Cer(t34:2)	$[M+H]^+$
248	794.5458	794.5460	0.3	PE(O-38:3)	$[M+K]^+$
249	794.5835	794.5824	1.4	CerP(d44:2)	$[M+K]^+$
250	794.6106	794.6116	1.3	HexCer(t38:1)	$[M+Na]^+$
251	795.4943	795.4937	0.8	PG(O-36:4)	$[M+K]^+$
252	795.5305	795.5301	0.5	PA(40:2)	$[M+K]^+$
253	795.5497	795.5510	1.6	PA(40:2(OH))	$[M+Na]^+$
254	796.5255	796.5253	0.3	PC(34:2)	$[M+K]^+$
255	796.5617	796.5617	< 0.1	PE(O-38:2)	$[M+K]^+$
256	796.5846	796.5851	0.6	PE(40:4)	$[M+H]^+$
257	797.5099	797.5093	0.8	PG(O-36:3)	$[M+K]^+$
258	797.5291	797.5303	1.5	LBPA(36:2)	$[M+Na]^+$
259	797.5655	797.5667	1.5	PA(40:1(OH))	$[M+Na]^+$
260	797.5932	797.5933	0.1	SM(d38:1)	$[M+K]^+$
261	798.5410	798.5410	< 0.1	PC(34:1)	$[M+K]^+$
262	798.5965	798.5983	2.3	PE(38:0)	$[M+Na]^+$
263	799.4085	799.4063	2.8	SQDG(30:3)	$[M+K]^+$
264	799.5444	799.5460	2.0	PG(36:1)	$[M+Na]^+$
265	800.4617	800.4627	1.2	PE(38:7)	$[M+K]^+$
266	800.5576	800.5566	1.2	PC(34:0)	$[M+K]^+$
267	800.6162	800.6164	0.2	PE(40:2)	$[M+H]^+$

268	801.4236	801.4220	2.0	SQDG(30:2)	$[M+K]^+$
269	801.5432	801.5430	0.2	TG(46:8)	$[M+K]^+$
270	801.5606	801.5616	1.2	PG(36:0)	$[M+Na]^+$
271	802.4784	802.4784	< 0.1	PE(38:6)	$[M+K]^+$
272	802.6321	802.6320	0.1	PE(40:1)	$[M+H]^+$
273	803.4385	803.4376	1.1	SQDG(30:1)	$[M+K]^+$
274	803.4593	803.4599	0.7	PPA(36:2)	$[M+Na]^+$
275	803.4818	803.4835	2.1	PG(34:1(OH))	$[M+K]^+$
276	803.5594	803.5586	1.0	TG(46:7)	$[M+K]^+$
277	803.5658	803.5668	1.2	MGDG(38:6)	$[M+H]^+$
278	804.4417	804.4424	0.9	PI-Cer(t32:2)	$[M+K]^+$
279	804.4923	804.4940	2.1	PE(38:5)	$[M+K]^+$
280	804.5512	804.5514	0.2	PC(36:4)	$[M+Na]^+$
281	804.6111	804.6113	0.2	PC(36:1(OH))	$[M+H]^+$
282	805.4547	805.4533	1.7	SQDG(30:0)	$[M+K]^+$
283	805.4957	805.4944	1.6	DGDG(26:2)	$[M+H]^+$
284	806.4573	806.4580	0.9	PI-Cer(t32:1)	$[M+K]^+$
285	806.5089	806.5097	1.0	PE(38:4)	$[M+K]^+$
286	806.5697	806.5694	0.4	PC(38:6)	$[M+H]^+$
287	806.6474	806.6480	0.7	HexCer(d40:1)	$[M+Na]^+$
288	807.4343	807.4348	0.6	SQDG(34:8)	$[M+H]^+$
289	807.5122	807.5100	2.7	DGDG(26:1)	$[M+H]^+$
290	808.4730	808.4737	0.9	PI-Cer(t32:0)	$[M+K]^+$
291	808.4869	808.4888	2.4	PE(40:9)	$[M+Na]^+$
292	808.5677	808.5698	2.6	PI-Cer(d36:1)	$[M+H]^+$
293	808.5839	808.5851	1.5	PC(38:5)	$[M+H]^+$
294	809.4498	809.4504	0.7	SQDG(34:7)	$[M+H]^+$
295	809.5713	809.5691	2.7	PG(O-40:6)	$[M+H]^+$
296	809.6506	809.6507	0.1	SM(d40:1)	$[M+Na]^+$
297	810.4334	810.4318	2.0	PS(34:4(OH))	$[M+K]^+$
298	810.5409	810.5410	0.1	PE(38:2)	$[M+K]^+$
299	810.5990	810.5983	0.9	PC(36:1)	$[M+Na]^+$
300	810.6813	810.6817	0.5	HexCer(d42:2)	$[M+H]^+$
301	811.4507	811.4521	1.7	PG(38:9)	$[M+Na]^+$
302	811.4677	811.4675	0.2	PA(42:8)	$[M+K]^+$
303	811.5850	811.5847	0.4	PG(O-40:5)	$[M+H]^+$
304	812.4981	812.4991	1.2	PE(P-40:7)	$[M+K]^+$
305	812.5195	812.5201	0.7	PE(40:7)	$[M+Na]^+$
306	812.5399	812.5412	1.6	PS(36:1)	$[M+Na]^+$
307	812.5568	812.5566	0.2	PE(38:1)	$[M+K]^+$
308	812.5908	812.5930	2.7	CerP(t44:1)	$[M+K]^+$
309	813.4834	813.4831	0.4	PA(42:7)	$[M+K]^+$
310	813.5017	813.5041	3.0	PA(42:7(OH))	$[M+Na]^+$
311	813.5229	813.5252	2.8	PG(36:2(OH))	$[M+Na]^+$
312	813.6841	813.6844	0.4	SM(d42:2)	$[M+H]^+$

313	814.5151	814.5147	0.5	PE(P-40:6)	$[M+K]^+$
314	814.5358	814.5359	0.1	PC(34:1(OH))	$[M+K]^+$
315	814.5556	814.5569	1.6	PS(36:0)	$[M+Na]^+$
316	814.6318	814.6320	0.2	PC(38:2)	$[M+H]^+$
317	815.5186	815.5197	1.3	PA(42:6(OH))	$[M+Na]^+$
318	815.5393	815.5409	2.0	PG(36:1(OH))	$[M+Na]^+$
319	815.6998	815.7000	0.2	SM(d42:1)	$[M+H]^+$
320	816.4941	816.4940	0.1	PC(36:6)	$[M+K]^+$
321	816.5290	816.5304	1.7	PE(O-40:6)	$[M+K]^+$
322	816.6476	816.6477	0.1	PC(38:1)	$[M+H]^+$
323	817.5142	817.5144	0.2	PA(42:5)	$[M+K]^+$
324	818.5101	818.5097	0.5	PC(36:5)	$[M+K]^+$
325	818.5448	818.5460	1.5	PE(O-40:5)	$[M+K]^+$
326	820.5255	820.5253	0.2	PC(36:4)	$[M+K]^+$
327	820.5853	820.5851	0.2	PE(42:6)	$[M+H]^+$
328	820.6057	820.6062	0.6	PS(38:0)	$[M+H]^+$
329	821.5289	821.5303	1.7	PG(38:4)	$[M+Na]^+$
330	821.5888	821.5902	1.7	PG(38:1(OH))	$[M+H]^+$
331	822.5410	822.5410	< 0.1	PC(36:3)	$[M+K]^+$
332	822.6215	822.6219	0.5	PS(O-38:0(OH))	$[M+H]^+$
333	822.6425	822.6429	0.5	HexCer(t40:1)	$[M+Na]^+$
334	823.4081	823.4063	2.2	SQDG(32:5)	$[M+K]^+$
335	823.5272	823.5272	< 0.1	PA(46:11)	$[M+H]^+$
336	823.5448	823.5460	1.5	PG(38:3)	$[M+Na]^+$
337	823.5622	823.5614	1.0	PA(42:2)	$[M+K]^+$
338	823.6080	823.6090	1.2	SM(d40:2)	$[M+K]^+$
339	824.4608	824.4627	2.3	PE(40:9)	$[M+K]^+$
340	824.5566	824.5566	< 0.1	PC(36:2)	$[M+K]^+$
341	824.6159	824.6164	0.6	PE(42:4)	$[M+H]^+$
342	824.6363	824.6376	1.6	HexCer(d40:0)	$[M+K]^+$
343	824.6570	824.6586	1.9	HexCer(t40:0)	$[M+Na]^+$
344	825.4238	825.4220	2.2	SQDG(32:4)	$[M+K]^+$
345	825.4640	825.4631	1.1	DGDG(28:6)	$[M+H]^+$
346	825.5599	825.5616	2.1	PG(38:2)	$[M+Na]^+$
347	825.6237	825.6246	1.1	SM(d40:1)	$[M+K]^+$
348	826.4766	826.4784	2.2	PE(40:8)	$[M+K]^+$
349	826.4995	826.4995	< 0.1	PS(36:2)	$[M+K]^+$
350	826.5721	826.5721	< 0.1	PE(P-42:6)	$[M+Na]^+$
351	827.4396	827.4376	2.4	SQDG(32:3)	$[M+K]^+$
352	827.4800	827.4787	1.6	DGDG(28:5)	$[M+H]^+$
353	827.5755	827.5773	2.2	PG(38:1)	$[M+Na]^+$
354	827.6401	827.6403	0.2	SM(d40:0)	$[M+K]^+$
355	828.4925	828.4940	1.8	PE(40:7)	$[M+K]^+$
356	828.5145	828.5150	0.6	PE(40:7(OH))	$[M+Na]^+$
357	828.5520	828.5515	0.6	PE(38:1(OH))	$[M+K]^+$

358	828.6453	828.6453	< 0.1	PE(O-40:0(OH))	$[M+Na]^+$
359	829.4547	829.4533	1.7	SQDG(32:2)	$[M+K]^+$
360	829.4960	829.4944	1.9	DGDG(28:4)	$[M+H]^+$
361	829.5750	829.5743	0.8	TG(48:8)	$[M+K]^+$
362	830.5099	830.5097	0.2	PE(40:6)	$[M+K]^+$
363	830.5286	830.5306	2.4	PE(40:6(OH))	$[M+Na]^+$
364	830.5578	830.5600	2.6	LacCer(d30:0)	$[M+Na]^+$
365	830.5672	830.5672	< 0.1	PE(38:0(OH))	$[M+K]^+$
366	830.6266	830.6269	0.4	PC(38:2(OH))	$[M+H]^+$
367	830.6634	830.6633	0.1	PE(42:1)	$[M+H]^+$
368	831.4342	831.4348	0.7	SQDG(36:10)	$[M+H]^+$
369	831.4695	831.4689	0.7	SQDG(32:1)	$[M+K]^+$
370	831.4918	831.4937	2.3	PA(42:6(OH))	$[M+K]^+$
371	831.5132	831.5148	1.9	PG(36:1(OH))	$[M+K]^+$
372	832.4718	832.4737	2.3	PI-Cer(t34:2)	$[M+K]^+$
373	832.5101	832.5099	0.2	PS(38:5)	$[M+Na]^+$
374	832.5833	832.5827	0.7	PC(38:4)	$[M+Na]^+$
375	832.6430	832.6426	0.5	PC(38:1(OH))	$[M+H]^+$
376	832.6628	832.6637	1.1	HexCer(d42:2)	$[M+Na]^+$
377	833.4502	833.4504	0.2	SQDG(36:9)	$[M+H]^+$
378	833.4855	833.4846	1.1	SQDG(32:0)	$[M+K]^+$
379	833.5259	833.5257	0.2	DGDG(28:2)	$[M+H]^+$
380	833.6485	833.6507	2.6	SM(d42:3)	$[M+Na]^+$
381	834.5400	834.5410	1.2	PE(40:4)	$[M+K]^+$
382	834.6012	834.6007	0.6	PC(40:6)	$[M+H]^+$
383	834.6788	834.6793	0.6	HexCer(d42:1)	$[M+Na]^+$
384	835.4658	835.4661	0.4	SQDG(36:8)	$[M+H]^+$
385	835.5434	835.5413	2.5	DGDG(28:1)	$[M+H]^+$
386	835.6656	835.6663	0.8	SM(d42:2)	$[M+Na]^+$
387	836.4970	836.4955	1.8	SHexCer(t34:0)	$[M+K]^+$
388	836.5180	836.5201	2.5	PE(42:9)	$[M+Na]^+$
389	836.5996	836.6011	1.8	PI-Cer(d38:1)	$[M+H]^+$
390	836.6125	836.6140	1.8	PC(38:2)	$[M+Na]^+$
391	836.6370	836.6375	0.6	DGTA(40:5)	$[M+Na]^+$
392	837.6407	837.6426	2.3	MGDG(38:0)	$[M+Na]^+$
393	837.6825	837.6820	0.6	SM(d42:1)	$[M+Na]^+$
394	838.4398	838.4420	2.6	PE(40:10(OH))	$[M+K]^+$
395	838.5727	838.5723	0.5	PE(40:2)	$[M+K]^+$
396	838.6157	838.6168	1.3	PI-Cer(d38:0)	$[M+H]^+$
397	838.6304	838.6296	1.0	PC(38:1)	$[M+Na]^+$
398	839.4988	839.4988	< 0.1	PA(44:8)	$[M+K]^+$
399	840.5511	840.5514	0.4	PE(42:7)	$[M+Na]^+$
400	840.5878	840.5878	< 0.1	PC(P-40:6)	$[M+Na]^+$
401	840.6227	840.6243	1.9	CerP(t46:1)	$[M+K]^+$
402	840.6328	840.6325	0.4	HexCer(t40:0)	$[M+K]^+$

403	841.5544	841.5565	2.5	PG(38:2(OH))	$[M+Na]^+$
404	841.6177	841.6195	2.1	SM(t40:1)	$[M+K]^+$
405	842.5100	842.5097	0.4	PC(38:7)	$[M+K]^+$
406	842.5669	842.5670	0.1	PE(42:6)	$[M+Na]^+$
407	842.6381	842.6399	2.1	CerP(t46:0)	$[M+K]^+$
408	842.6623	842.6633	1.2	PC(40:2)	$[M+H]^+$
409	843.5134	843.5147	1.5	PG(40:7)	$[M+Na]^+$
410	843.5702	843.5722	2.4	PG(38:1(OH))	$[M+Na]^+$
411	843.6352	843.6352	< 0.1	SM(t40:0)	$[M+K]^+$
412	844.5076	844.5101	3.0	PI-Cer(d36:2)	$[M+K]^+$
413	844.5253	844.5253	< 0.1	PC(38:6)	$[M+K]^+$
414	844.6783	844.6790	0.8	PC(40:1)	$[M+H]^+$
415	845.5287	845.5303	1.9	PG(40:6)	$[M+Na]^+$
416	846.4659	846.4682	2.7	PS(38:6)	$[M+K]^+$
417	846.5255	846.5257	0.2	PI-Cer(d36:1)	$[M+K]^+$
418	846.5409	846.5410	0.1	PC(38:5)	$[M+K]^+$
419	846.6008	846.6007	0.1	PE(44:7)	$[M+H]^+$
420	846.6213	846.6219	0.7	PS(40:1)	$[M+H]^+$
421	847.4061	847.4063	0.2	SQDG(34:7)	$[M+K]^+$
422	847.5456	847.5460	0.5	PG(40:5)	$[M+Na]^+$
423	847.6639	847.6663	2.8	PE-Cer(d46:3)	$[M+Na]^+$
424	848.4815	848.4837	2.6	PE(42:11(OH))	$[M+Na]^+$
425	848.5567	848.5566	0.1	PC(38:4)	$[M+K]^+$
426	848.6163	848.6164	0.1	PE(44:6)	$[M+H]^+$
427	848.6370	848.6375	0.6	PS(40:0)	$[M+H]^+$
428	848.6576	848.6586	1.2	HexCer(t42:2)	$[M+Na]^+$
429	849.4240	849.4220	2.4	SQDG(34:6)	$[M+K]^+$
430	849.5600	849.5616	1.9	PG(40:4)	$[M+Na]^+$
431	849.6214	849.6215	0.1	PG(40:1(OH))	$[M+H]^+$
432	850.4764	850.4784	2.4	PE(42:10)	$[M+K]^+$
433	850.5710	850.5709	0.1	SHexCer(t38:2)	$[M+H]^+$
434	850.6520	850.6532	1.4	PS(O-40:0(OH))	$[M+H]^+$
435	850.6736	850.6742	0.7	HexCer(t42:1)	$[M+Na]^+$
436	851.4392	851.4376	1.9	SQDG(34:5)	$[M+K]^+$
437	851.4800	851.4810	1.2	PGP(34:1)	$[M+Na]^+$
438	851.5587	851.5585	0.2	PA(48:11)	$[M+H]^+$
439	851.5758	851.5773	1.8	PG(40:3)	$[M+Na]^+$
440	851.6392	851.6403	1.3	SM(d42:2)	$[M+K]^+$
441	852.4918	852.4940	2.6	PE(42:9)	$[M+K]^+$
442	852.5130	852.5150	2.3	PE(42:9(OH))	$[M+Na]^+$
443	852.5876	852.5879	0.4	PC(38:2)	$[M+K]^+$
444	852.6448	852.6453	0.6	PE(42:1)	$[M+Na]^+$
445	852.6825	852.6817	0.9	CerP(t48:1)	$[M+Na]^+$
446	853.4185	853.4167	2.1	SQDG(36:10)	$[M+Na]^+$
447	853.4952	853.4944	0.9	DGDG(30:6)	$[M+H]^+$

448	853.5909	853.5929	2.3	PG(40:2)	$[M+Na]^+$
449	853.6558	853.6559	0.1	SM(d42:1)	$[M+K]^+$
450	854.4977	854.4967	1.2	PS(42:11)	$[M+H]^+$
451	854.5303	854.5306	0.4	PE(42:8(OH))	$[M+Na]^+$
452	854.5675	854.5672	0.4	PE(40:2(OH))	$[M+K]^+$
453	854.6035	854.6036	0.1	PC(38:1)	$[M+K]^+$
454	854.6389	854.6399	1.2	PE(O-42:1)	$[M+K]^+$
455	854.6594	854.6609	1.8	PE(42:0)	$[M+Na]^+$
456	855.4913	855.4937	2.8	PA(44:8(OH))	$[M+K]^+$
457	855.6067	855.6086	2.2	PG(40:1)	$[M+Na]^+$
458	856.5241	856.5253	1.4	PE(42:7)	$[M+K]^+$
459	856.5457	856.5463	0.7	PE(42:7(OH))	$[M+Na]^+$
460	856.5828	856.5828	< 0.1	PE(40:1(OH))	$[M+K]^+$
461	857.4505	857.4480	2.9	SQDG(36:8)	$[M+Na]^+$
462	858.5044	858.5044	< 0.1	PE(44:12)	$[M+Na]^+$
463	858.5248	858.5256	0.9	PS(40:6)	$[M+Na]^+$
464	858.5416	858.5410	0.7	PE(42:6)	$[M+K]^+$
465	858.5609	858.5619	1.2	PE(42:6(OH))	$[M+Na]^+$
466	858.5891	858.5913	2.6	LacCer(d32:0)	$[M+Na]^+$
467	859.5450	859.5461	1.3	PG(38:1(OH))	$[M+K]^+$
468	860.5198	860.5201	0.3	PE(44:11)	$[M+Na]^+$
469	860.6148	860.6140	0.9	PC(40:4)	$[M+Na]^+$
470	861.5175	861.5159	1.9	SQDG(34:0)	$[M+K]^+$
471	862.5338	862.5357	2.2	PE(44:10)	$[M+Na]^+$
472	862.6160	862.6168	0.9	PI-Cer(d40:2)	$[M+H]^+$
473	864.4552	864.4576	2.8	PE(42:11(OH))	$[M+K]^+$
474	864.6315	864.6324	1.0	PI-Cer(d40:1)	$[M+H]^+$
475	866.6474	866.6481	0.8	PI-Cer(d40:0)	$[M+H]^+$
476	867.4537	867.4549	1.4	PGP(34:1)	$[M+K]^+$
477	867.6585	867.6603	2.1	PA(O-46:1)	$[M+K]^+$
478	868.5253	868.5253	< 0.1	PC(40:8)	$[M+K]^+$
479	868.6190	868.6191	0.1	PC(P-42:6)	$[M+Na]^+$
480	868.6531	868.6556	2.9	CerP(t48:1)	$[M+K]^+$
481	868.6623	868.6638	1.7	HexCer(t42:0)	$[M+K]^+$
482	869.6496	869.6508	1.4	SM(t42:1)	$[M+K]^+$
483	870.4661	870.4682	2.4	PS(40:8)	$[M+K]^+$
484	870.5409	870.5410	0.1	PC(40:7)	$[M+K]^+$
485	870.6938	870.6946	0.9	PC(42:2)	$[M+H]^+$
486	871.5443	871.5460	2.0	PG(42:7)	$[M+Na]^+$
487	872.5566	872.5566	< 0.1	PC(40:6)	$[M+K]^+$
488	873.4235	873.4220	1.7	SQDG(36:8)	$[M+K]^+$
489	873.5600	873.5616	1.8	PG(42:6)	$[M+Na]^+$
490	874.4775	874.4784	1.0	PE(44:12)	$[M+K]^+$
491	874.4993	874.4993	< 0.1	PE(44:12(OH))	$[M+Na]^+$
492	874.5550	874.5570	2.3	PI-Cer(d38:1)	$[M+K]^+$

493	875.5028	875.5045	1.9	LPIP(32:1)	$[M+H]^+$
494	876.5129	876.5150	2.4	PE(44:11(OH))	$[M+Na]^+$
495	876.5636	876.5631	0.6	SHexCer(d38:0)	$[M+K]^+$
496	876.5878	876.5879	0.1	PC(40:4)	$[M+K]^+$
497	877.5912	877.5929	1.9	PG(42:4)	$[M+Na]^+$
498	878.5073	878.5097	2.7	PE(44:10)	$[M+K]^+$
499	878.5280	878.5306	3.0	PE(44:10(OH))	$[M+Na]^+$
500	879.5107	879.5100	0.8	DGDG(32:7)	$[M+H]^+$
501	880.5233	880.5253	2.3	PE(44:9)	$[M+K]^+$
502	880.5449	880.5463	1.6	PE(44:9(OH))	$[M+Na]^+$
503	880.6184	880.6178	0.7	SHexCer(t40:1)	$[M+H]^+$
504	881.6219	881.6242	2.6	PG(42:2)	$[M+Na]^+$
505	882.6344	882.6347	0.3	PE(P-46:6)	$[M+Na]^+$
506	883.6376	883.6399	2.6	PG(42:1)	$[M+Na]^+$
507	888.5509	888.5514	0.6	PE(46:11)	$[M+Na]^+$
508	892.5255	892.5253	0.2	PC(42:10)	$[M+K]^+$
509	892.6612	892.6637	2.8	PI-Cer(d42:1)	$[M+H]^+$
510	895.6905	895.6916	1.2	PA(O-48:1)	$[M+K]^+$
511	896.6498	896.6504	0.7	PC(P-44:6)	$[M+Na]^+$
512	897.4846	897.4865	2.1	LPIP(32:1)	$[M+Na]^+$
513	898.4893	898.4900	0.8	MIPC(t28:0)	$[M+Na]^+$
514	898.5723	898.5723	< 0.1	PC(42:7)	$[M+K]^+$
515	899.5759	899.5773	1.6	PG(44:7)	$[M+Na]^+$
516	904.5228	904.5253	2.8	PE(46:11)	$[M+K]^+$
517	904.5456	904.5463	0.8	PE(46:11(OH))	$[M+Na]^+$
518	905.4944	905.4941	0.3	PI(P-38:6)	$[M+K]^+$
519	907.5414	907.5413	0.1	DGDG(34:7)	$[M+H]^+$
520	908.6496	908.6491	0.6	SHexCer(t42:1)	$[M+H]^+$
521	909.5461	909.5463	0.2	Glc-GP(38:4)	$[M+Na]^+$
522	909.6529	909.6555	2.9	PG(44:2)	$[M+Na]^+$
523	909.6696	909.6709	1.4	PA(48:1)	$[M+K]^+$
524	910.6657	910.6660	0.3	PE(P-48:6)	$[M+Na]^+$
525	911.6688	911.6712	2.6	PG(44:1)	$[M+Na]^+$
526	913.4586	913.4604	2.0	LPIP(32:1)	$[M+K]^+$
527	915.5989	915.6015	2.8	DGDG(32:0)	$[M+Na]^+$
528	916.5252	916.5253	0.1	PC(44:12)	$[M+K]^+$
529	917.4674	917.4659	1.6	DGDG(32:7)	$[M+K]^+$
530	919.4706	919.4708	0.2	LPIP(34:4)	$[M+Na]^+$
531	919.6894	919.6916	2.4	PA(O-50:3)	$[M+K]^+$
532	920.5180	920.5202	2.4	PE(46:11(OH))	$[M+K]^+$
533	920.6930	920.6950	2.2	PI-Cer(d44:1)	$[M+H]^+$
534	921.5143	921.5124	2.1	PI(40:9(OH))	$[M+H]^+$
535	923.5043	923.5045	0.2	PIP(O-36:5)	$[M+H]^+$
536	925.5199	925.5202	0.3	CL(36:4)	$[M+H]^+$
537	926.5237	926.5213	2.6	MIPC(t30:0)	$[M+Na]^+$

538	931.5286	931.5307	2.3	PI(40:7)	$[M+Na]^+$
539	935.4442	935.4448	0.6	LPIP(34:4)	$[M+K]^+$
540	935.4967	935.4950	1.8	SQDG(42:11)	$[M+Na]^+$
541	936.6810	936.6804	0.6	SHexCer(t44:1)	$[M+H]^+$
542	937.7005	937.7022	1.8	PA(50:1)	$[M+K]^+$
543	941.4915	941.4917	0.2	PIP(O-34:1)	$[M+K]^+$
544	945.4870	945.4865	0.5	PIP(O-36:5)	$[M+Na]^+$
545	947.5024	947.5021	0.3	CL(36:4)	$[M+Na]^+$
546	959.4448	959.4448	< 0.1	PIP(P-36:5)	$[M+K]^+$
547	961.4604	961.4604	< 0.1	PIP(O-36:5)	$[M+K]^+$
548	961.5119	961.5106	1.4	SQDG(44:12)	$[M+Na]^+$
549	963.4762	963.4761	0.1	CL(36:4)	$[M+K]^+$
550	965.6161	965.6172	1.1	DGDG(36:3)	$[M+Na]^+$
551	969.4850	969.4865	1.5	PIP(P-38:6)	$[M+Na]^+$
552	969.5489	969.5465	2.5	PI(40:4(OH))	$[M+K]^+$
553	978.5228	978.5216	1.2	CDP-DG(34:2)	$[M+H]^+$
554	981.5897	981.5911	1.4	DGDG(36:3)	$[M+K]^+$
555	982.5922	982.5934	1.2	PS(48:8)	$[M+K]^+$
556	987.4758	987.4761	0.3	PIP(O-38:6)	$[M+K]^+$
557	987.5614	987.5628	1.4	SQDG(44:7)	$[M+K]^+$
558	989.5426	989.5419	0.7	SQDG(46:12)	$[M+Na]^+$
559	992.4220	992.4227	0.7	M(IP)2C(t20:0)	$[M+H]^+$

Supporting Table 3: Established protocol for hematoxylin and eosin staining. Tissue sections were submerged in solvents for the dedicated time.

**Solvent**	**Duration**
100 % ethanol	2 min
70 % ethanol	2 min
40 % ethanol	2 min
Aqua dest.	2 min
Hematoxylin	12 min
Tap water	10 min
1 % eosin Y	1 min
Aqua dest.	2 min
40 % ethanol	2 min
70 % ethanol	2 min
100 % ethanol	2 min
Xylene	2 min

References

1. Liebisch, G.; Vizcaíno, J.A.; Köfeler, H.; Trötzmüller, M.; Griffiths, W.J.; Schmitz, G.; Spener, F.; Wakelam, M.J.O. Shorthand notation for lipid structures derived from mass spectrometry. *Journal of Lipid Research* **2013**, *54*, 1523–1530, doi:10.1194/jlr.M033506.
2. Liebisch, G.; Fahy, E.; Aoki, J.; Dennis, E.A.; Durand, T.; Ejsing, C.S.; Fedorova, M.; Feussner, I.; Griffiths, W.J.; Köfeler, H.; et al. Update on LIPID MAPS classification, nomenclature, and shorthand notation for MS-derived lipid structures. *Journal of Lipid Research* **2020**, *61*, 1539–1555, doi:10.1194/jlr.S120001025.
